# Enriching the endophytic bacterial microbiota of *Ginkgo* roots

**DOI:** 10.3389/fmicb.2023.1163488

**Published:** 2023-04-17

**Authors:** Shuangfei Zhang, Chongran Sun, Xueduan Liu, Yili Liang

**Affiliations:** ^1^School of Minerals Processing and Bioengineering, Central South University, Changsha, China; ^2^Key Laboratory of Biometallurgy, Ministry of Education, Changsha, Hunan, China

**Keywords:** *Ginkgo biloba*, endophytic bacteria, enrichment culture, plant microbiome, root endosphere

## Abstract

Bacterial endophytes of *Ginkgo* roots take part in the secondary metabolic processes of the fossil tree and contribute to plant growth, nutrient uptake, and systemic resistance. However, the diversity of bacterial endophytes in *Ginkgo* roots is highly underestimated due to the lack of successful isolates and enrichment collections. The resulting culture collection contains 455 unique bacterial isolates representing 8 classes, 20 orders, 42 families, and 67 genera from five phyla: Actinobacteria, Bacteroidetes, Firmicutes, Proteobacteria, and Deinococcus-Thermus, using simply modified media (a mixed medium without any additional carbon sources [MM)] and two other mixed media with separately added starch [GM] and supplemented glucose [MSM]). A series of plant growth-promoting endophytes had multiple representatives within the culture collection. Moreover, we investigated the impact of refilling carbon sources on enrichment outcomes. Approximately 77% of the natural community of root-associated endophytes were predicted to have successfully cultivated the possibility based on a comparison of the 16S rRNA gene sequences between the enrichment collections and the *Ginkgo* root endophyte community. The rare or recalcitrant taxa in the root endosphere were mainly associated with Actinobacteria, Alphaproteobacteria, Blastocatellia, and Ktedonobacteria. By contrast, more operational taxonomic units (OTUs) (0.6% in the root endosphere) became significantly enriched in MM than in GM and MSM. We further found that the bacterial taxa of the root endosphere had strong metabolisms with the representative of aerobic chemoheterotrophy, while the functions of the enrichment collections were represented by the sulfur metabolism. In addition, the co-occurrence network analysis suggested that the substrate supplement could significantly impact bacterial interactions within the enrichment collections. Our results support the fact that it is better to use the enrichment to assess the cultivable potential and the interspecies interaction as well as to increase the detection/isolation of certain bacterial taxa. Taken together, this study will deepen our knowledge of the indoor endophytic culture and provide important insights into the substrate-driven enrichment.

## 1. Introduction

Endophytic bacteria in roots are an important component of plant endophytes. Within the root endosphere, bacterial concentrations can reach 10^4^ of 10^5^ cell/g of root material and sometimes even reach 10^8^ cells/g of root (Bulgarelli et al., 2013; Utturkar et al., 2016), which is much lower than that estimated for 10^9^ cells/g of rhizosphere soil (Bulgarelli et al., 2013). Most of the bacterial endophytes are derived from the rhizosphere or phyllosphere, while some of them may be seedborne (Ryan et al., 2008; Shakya et al., 2013; Papik et al., 2020). Generally, the roots show a surprising microbiome variation and the *in situ* bacterial communities are driven by the plant genotype involving both abiotic and biotic variables (i.e., environmental factors, plant–microbe interactions, and microbe–microbe interactions) (Miliute et al., 2015; Fitzpatrick et al., 2020; Compant et al., 2021; Attia et al., 2022). Even so, endophytic bacteria help their hosts by promoting plant fitness, suppressing pathogens, removing soil contaminants, solubilizing phosphate, or contributing assimilable nitrogen to plants (Lodewyckx et al., 2002; Rosenblueth and Martínez-Romero, 2006; Compant et al., 2010; Reinhold-Hurek and Hurek, 2011; Sánchez-López et al., 2018). In recent years, researchers have been showing a growing interest in developing the potential applications of bacterial endophytes for exploring the plant–microbe interactions and promoting the sustainable production of crops (Papik et al., 2020; Carper et al., 2021; Dwibedi et al., 2022; Raaijmakers and Kiers, 2022; Riva et al., 2022). However, as most of the bacterial endophytes closely related to plants are difficult to culture, their real diversities and interactions with their hosts are still unclear.

As observed in previous studies, useful information has been provided by culture-dependent and culture-independent methodologies for microbial diversity in plant-associated ecosystems (Leff et al., 2015; Carper et al., 2021). Among them, the basic cultivation-based approaches are indispensable and popular, which can help obtain both the phenotypes and genotypes of microbes (Zhang and Zhang, 2022). Specifically, cultivation can successfully isolate uncultured bacteria from the plant microbiome, which can be further exploited to improve plant holobiont's health and promote plant production (Riva et al., 2022). Thus far, although a certain number of previously uncultured species have been successfully isolated, it is still a major challenge that most of the plant-associated microbes do not have a cultured representative. As reported, some microbial species may fail to grow because of the auxotrophic nature of microbes with minimal genomes and restricted anabolic capacities (Hug et al., 2016) as well as the oligo-/prototrophic nature of microbes with large genomes, complex metabolism, and restricted replication mechanisms (Sarhan et al., 2019). To address these challenges, a series of innovative cultivation strategies were employed previously to isolate microbes, such as a relatively simple method using various media (Carper et al., 2021), simply modified media (Nishioka and Tamaki, 2022), mixed culture using plant-based culture media (PCM) (Sarhan et al., 2019), and differential centrifugation combined with enzymatic hydrolysis (Jiao et al., 2006). These aforementioned strategies indicate high feasibility for the application of indoor enrichment methods for exploring the diversity of plant-associated bacteria and for conducting a metagenomic analysis to study their sequencing patterns (Ikeda et al., 2009; Sarhan et al., 2019).

Although cultivation strategies have transferred from axenic to mixed cultures (Nai and Meyer, 2018) and some bacterial species display a mixed-culture dependence (Kaeberlein et al., 2002; Stewart Eric, 2012), it may be difficult to subject the endophytic microorganisms obtained from an enrichment culture to practical implementation. The indoor enrichment culture method is mainly aimed at obtaining functionally valuable microorganisms with enormous quantities of biomass under common culture conditions, not just their ecological significance. As reported by Mu et al. (2018, 2021), if one isolate could be achieved by the preferred carbon/nutrient sources, one like the dormancy should be theoretically able to grow. The host plant provides the endophytic bacteria with a rich habitat (Sánchez-López et al., 2018) and the carbon sources may play a key factor in influencing the endophytes. In addition to glucose, the endophytes can metabolize the starch in plant tissues (Pundir et al., 2014). A recent study illustrates the point that many endophytes with a positive amylase activity were isolated on starch agar plates (Singh et al., 2022). In addition, considering that the living environments of bacterial endophytes are liquid and some species may not grow on solid media, enrichment in liquid media is necessary. Thus, studies regarding enriching functional endophytes from plants under common culture conditions and using the drive of different substrates are worthy to be further characterized.

The studies on woody plants that are accessible mainly cover species that are important for plant–microbe interactions or for phytoremediation, such as *Ginkgo biloba* (Yuan et al., 2019; Toghueo, 2020) and *Populus* spp. (Gottel et al., 2011; Utturkar et al., 2016; Carper et al., 2021). Compared with *Populus* spp., it is worth noting that the diversity of the bacterial endophytes in *Ginkgo* roots is highly underestimated (Toghueo, 2020; Zou et al., 2021). *Ginkgo biloba* is one ancient medicinal plant and is of great evolutionary significance, which contains abundant endophytes and diverse secondary metabolites (Wang et al., 2017; Yuan et al., 2019; Zhao et al., 2019). In addition, this tree hosts abundant and diverse endophytic microbes. Given different *Ginkgo* tissues, the bacterial endophytes in the root are significantly more abundant than those in the leaf and stem (Shehata et al., 2021). Bacterial endophytes that colonize with *Ginkgo* trees contribute to plant growth, nutrient uptake, and systemic resistance (Yuan et al., 2019). In addition, one of our studies indicates that many root endophytes of *Ginkgo biloba* participate in secondary metabolic processes and exchange genetic information with their host trees (Zou et al., 2021).

In this study, we focused on enriching the bacterial endophytes of *Ginkgo* roots using mixed low-nutrient media with the supplement of glucose or starch. Using pure culture and amplicon sequencing, we aimed to (i) obtain some uncultured microbes and plant growth-promoting endophytes from the root endosphere of *Ginkgo* trees to show the potential of the enrichment culture, (ii) explore to what extent the indoor enrichment represents the bacterial diversity of the *in situ* root tissues, and (iii) assess the synergistic effects of co-culture of glucose and starch on the endophytic bacterial communities of *Ginkgo* roots. With the enrichment under different carbon sources, we will be able to reserve a large proportion of culturable bacterial endophytes without considering metagenome-assembled methods. This study will deepen our knowledge of the indoor endophytic culture and provide important insights into the substrate-driven enrichment.

## 2. Materials and methods

### 2.1. Sample collection and processing

The root tissues of *Ginkgo biloba* L. used in this study were collected in Linyi City, Shandong Province, China (Lat 34°36′34^′′^ N, Lon 118°12′8^′′^ E, with an altitude of 40 m) in October 2019. Six individuals, each at least 5 m apart, were randomly chosen to collect the roots according to a previous study (Chen et al., 2019). A few root tissues of the *Ginkgo* trees, which was 1 m deep underground and 1.5 m horizontally away for the trunk, were dug out and lightly shaken; then, for each individual tree, several root tissues of 2-cm diameter were cut off and mixed to make a composite sample. The roots of *Ginkgo* trees were packed into polyethylene bags, placed on ice packs in a cooler after collection, and immediately transported to the laboratory, where they were stored at 4°C until further processing. Rhizosphere soil was collected and processed as given in a previous study (Beckers et al., 2017). From each sample, the soil particles adhering to the roots were collected by lightly shaking the roots for 20 min and regarded as the rhizosphere soil. The root samples were sonicated for 10 min in an ultrasonic bath cleaner (Branson, CT, USA) (Richter-Heitmann et al., 2016). The root tissues were surface disinfected in 70% CH_3_CH_2_OH, NaClO, and sterile Millipore water, which is consistent with that given in a previous study (Beckers et al., 2017). To verify if surface sterilization were successful, the final washed water was inoculated onto potato dextrose agar (PDA) culture. Then, the surface-disinfected roots were stored until they to be sequenced (stored at −80°C after placing in liquid nitrogen for 5 min) and thereafter subjected to enrichment culture.

### 2.2. Enrichment culture and pure culture

Three media for enrichment culture were low-nutrient media on the basis of improved minimal medium (MM). The composition of MM was shown as follows: NH_4_NO_3_ (1.0 g·L^−1^), KH_2_PO_4_ (0.5 g·L^−1^), Na_2_HPO_4_ (1.5 g·L^−1^), NaCl (0.5 g·L^−1^), MgSO_4_·7H_2_O (0.2 g·L^−1^), FeCl_3_ (5.0 mg·L^−1^), (NH_4_)_2_MoO_4_ (1.0 mg·L^−1^), and horny coral skeletons (1.0 g·L^−1^). In addition, one medium named GM was based on MM with starch (2.0 g·L^−1^), while the other medium named MSM was prepared according to MM with 2.0 g·L^−1^ supplemented glucose. As previously reported (Raina et al., 2009), air-dried coral skeletons were crushed, diluted, and supplied to the culture media, which were used for the bacterial attachment in this study. During incubation, the pH was adjusted to 7.0 with 10% (*w*/*v*) NaHCO_3_ or 2% (*w*/*v*) KH_2_PO_4_. The incubation of enrichment culture was first performed at 25°C for 8 days in separate 300-mL sealed glass bottles (filled with 200 mL of liquid medium and 4.0 g·L^−1^ of root homogenates). Three bottles of each medium were chosen as the blank group to test the sterilizing effect. In detail, 3.0 g of the surface-sterilized root sample from each individual was ground in 6 mL of sterile water, equally divided into three parts, and, respectively, transferred into three liquid culture media, including MM, GM, and MSM, each replicated three times, to incubate for 8 days at 25°C. Then, half of the fermentative broth was transferred to the corresponding new media and cultured for another 2 days at 25°C; the subsequent fermentative broth was evenly mixed according to different root samples and sampled for DNA extraction. Due to the presence of plant tissues, the fungal biomass obviously existed in the mixed fermentative broth during the first 8 days of enrichment (Supplementary Figure S1). Thus, the two-step culture method could prevent the loss of rare taxa and the effect of endophytic fungi. To prevent the growth of phototrophic microorganisms, the whole enrichment culture was kept under reduced light exposure. The shaking speed was 150 rpm.

The pure culture method and the species identification were carried out in accordance with those given in our previous study (Zou et al., 2021) and the main difference between the two studies pertains to the media used in this study. The three media, MM, MSM, and GM, were supplemented with 15 g·L^−1^ of agarose and used to isolate endophytic bacteria. For culturing the bacterial endophytes, the mixed fermentative broth after the first 8 days of enrichment was used as an isolation source. Rhizosphere soil (10 g) was sufficiently dissolved in 100 mL of phosphate-buffered saline (PBS) using a magnetic stirrer (Shanghai Zhenrong Scientific Instrument Co., Ltd., China). Aliquots of 100 μL of each fermentative broth/solution were diluted serially 10-fold and an additional 100 μL of the three dilutions (10^−8^, 10^−9^, and 10^−10^ for the fermentative broth; 10^−7^, 10^−8^, and 10^−9^ for the soil solution) was spread onto MM, GM, and MSM agar plates. All plates were incubated at 25°C for the next 3 days to examine the microbial growth. The colonies having different morphological features were streaked individually onto their corresponding agar plates and incubated again for another 3 days at 25°C for growth. A single colony of each isolate was further cultured in its corresponding liquid media for 48 h to cultivate enough bacterial cells for cryopreservation (−80°C with the addition of 30% glycerol) and DNA extraction. Genomic DNA of each individual isolate was extracted by the CTAB method. The PCR amplification of each isolate's 16S rRNA gene was conducted using universal primers 27F and 1492R (Zou et al., 2021) and then sequenced at the Beijing Genomics Institute (BGI). The 16S rRNA gene sequence of each isolate was further analyzed using the BLASTn tool [National Center for Biotechnology Information (NCBI), USA] and presumptively identified on the bases of the top BLAST hits.

### 2.3. DNA extraction and amplicon sequencing

Before DNA extraction, six surface-sterilized root samples were ground in liquid nitrogen using sterile mortars and pestles, and 18 enrichment samples were centrifuged at low temperatures to collect bacterial cells. The total DNA of 200-mg ground *Ginkgo* roots was extracted using the CTAB method for each surface-sterilized root sample (Murray and Thompson, 1980), and 0.3 g of centrifugal bacterial cells was extracted with a DNA Isolation Kit (BioTeke, Beijing, China) according to manufacturer's instructions. The concentration and purity of DNA were measured using a NanoDrop ND-1000 spectrophotometer (NanoDrop Technologies, Wilmington, USA). The DNA quality was evaluated by 1.0% agarose gel electrophoresis with ethidium bromide (EB). The partially extracted DNA was diluted to a concentration of 1 ng/μL and then stored at −80°C until further processing. We used the diluted DNA as a template for PCR amplification of 16S rRNA genes with the HiFi HotStart ReadyMix (KAPA) and barcoded primers. For each extracted DNA sequence, V3–V4 variable regions of bacterial 16S rRNA genes were amplified using the universal primers 338 F (5′- ACTCCTACGGGAGGCAGCA-3′) and 806 R (5′- GGACTACHVGGGTWTCTAAT−3′). Sequencing was carried out on a MiSeq platform at the Guangzhou Magigen Biotechnology Co., Ltd. (Guangzhou, China). Detailed protocols were presented as the method described previously (Chen et al., 2019).

### 2.4. Taxonomic fingerprint during enrichment

The prophase amplicon sequences were processed following the same procedures previously described (Zhang et al., 2019). In brief, the sequencing raw data were analyzed using QIIME2 (Bolyen et al., 2019), USEARCH version 10.0 (Edgar, 2010), and in-house scripts (Zhang et al., 2018). The operational taxonomic unit (OTU) was selected on a similarity threshold of 97% using the UPARSE algorithm (Edgar, 2013). One representative sequence chosen from each OTU was assigned against the SILVA 132 database (Quast et al., 2013) to obtain the taxonomic information. Then, sequences <80% similarity to the reference sequence were identified as unclassified and removed from bacterial communities.

The relative abundance of individual taxon was calculated using the online tool MicrobiomeAnalyst (Chong et al., 2020). The core taxa prediction and alpha diversity were also calculated using MicrobiomeAnalyst. The alpha diversity was estimated by Observed, Chao1, Shannon, abundance-based coverage (ACE), Fisher, and Simpson index. Fisher's least significant difference (LSD) was used to calculate the significance of alpha diversity among different groups. Principal coordinate analysis (PCoA) and non-metric multidimensional scaling analysis (NMDS) based on the Bray–Curtis distance were used to estimate the heterogeneity in microbial community. The statistically significant differences in community composition were assessed using the permutational multivariate analysis of variance (PERMANOVA). All of the aforementioned and following analyses were performed using R (Version 4.1.2) with the “vegan” package.

In addition, to explore the differential OTU abundance and taxa, Wilcoxon's rank-sum tests according to OTUs with median relative abundance from each genotype >0.2% were performed. Meanwhile, the maximum FDR was set as 0.05 and the FDR-adjusted *P*-values were corrected for multiple comparisons tests. Manhattan plots were used to display the enrichment of OTUs based on their taxonomy. FAPROTAX version 1.1 (Louca et al., 2016) and Tax4Fun2 (Wemheuer et al., 2020) were used to predict the functional potential of bacterial communities in the *Ginkgo* roots and the indoor shakers. We compared the differences in function among them at a particular Kyoto Encyclopedia of Genes and Genomes (KEGG, https://www.kegg.jp/) pathway levels 1, 2, and 3. A *t*-test/analysis of variance (ANOVA) was applied to measure the significance of functions among different experimental groups.

### 2.5. Correlation analysis

To illustrate potential microbial interactions in the *Ginkgo* roots and the indoor shakers, OTU co-occurrence networks were reconstructed based on Spearman's correlations as described in a previous study (Hiraoka et al., 2020). The OTUs detected in more than 20% of samples and that represented > 0.1% of the total sequences were considered for the construction of the interspecies co-occurrence network. All pairwise Spearman's correlations (*r*) of those retained OTUs were measured using the “Hmisc” library in R. Only the robust (|*r*| > 0.8) and statistically significant (*P*-value < 0.01 after Bonferroni's correction) correlations were incorporated into the construction of co-occurrence networks. Then, subnetworks involved in more than two OTUs were retained, and the co-occurrence networks were visualized using Gephi 0.9.2 (https://gephi.org). Additionally, the Sparse Correlations Network Investigation for Compositional data algorithm (SparCC) network within MicrobiomeAnalyst was also used to calculate the significant correlations among bacterial taxa at family level (Friedman and Alm, 2012). Only the strong (correlation threshold > 0.3) and significant (*P*-value < 0.05) correlations were entered in the network plot.

### 2.6. Data availability

All sequencing amplicon sequence data associated with this study have been deposited in the National Center for Biotechnology Information (NCBI) Sequence Read Archive (SRA) database under Accession No. PRJNA889250.

## 3. Results

### 3.1. Composition and diversity of cultured bacteria from *Ginkgo* roots

**Figure 1:**
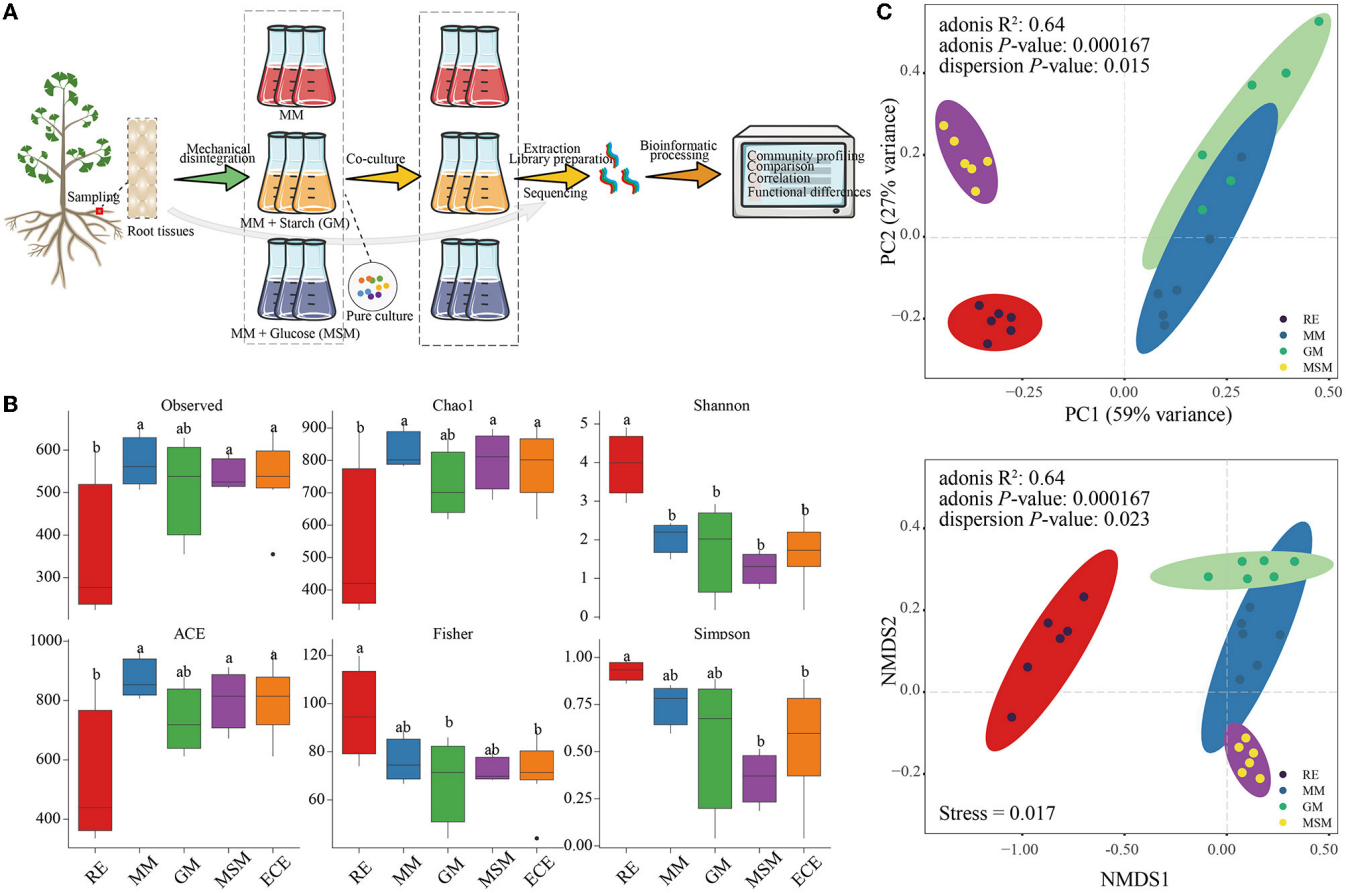
Schematic procedure of cultivating the endophytic bacteria of *Ginkgo* roots and the diversity of their bacterial communities. **(A)** A workflow enriching the endophytic bacteria of the *Ginkgo* root tissues. **(B)** Alpha diversity indices of bacterial communities in the *in situ* root tissues and the enrichment culture, including the Observed species, Chao1, Shannon, ACE, Fisher, and Simpson index. ECE represents the whole enrichment culture samples. **(C)** PCA and NMDS ordinations of the Bray–Curtis dissimilarity matrices with PERMANOVA, showing a significant variance of the bacterial community composition between the *in situ* root samples and enrichment collections. Table 1 presents a list of abbreviations of the groups used in the figure.

To obtain a large proportion of culturable bacterial endophytes of *Ginkgo* roots, an enrichment culture method using low-nutrient media containing root homogenates in the mixed culture was exploited (Figure 1A). Both the *Ginkgo* rhizosphere soil and the root endosphere were designated as isolate resources in an effort to increase as much diversity as possible. Totally, large (455) bacterial culture collections from *Ginkgo* roots were successfully isolated under common culture conditions. Among them, 320 and 135 pure bacterial cultures were obtained from the root endosphere and rhizosphere (Supplementary Table S1), respectively. The majority of the culture collections (70%) were obtained using GM, although other media were also applied to capture strain diversity. Based on the 16S rRNA gene sequence analysis, the diversity of the culture collections was determined to contain bacterial isolates representing 8 classes, 20 orders, 42 families, and 67 genera from five phyla: Actinobacteria, Bacteroidetes, Firmicutes, Proteobacteria, and Deinococcus-Thermus. The greatest number of collections is from six genera, such as *Bacillus* (38.2%, 174), *Paenibacillus* (8.8%, 40), *Streptomyces* (8.4%, 38), *Flavobacterium* (4.1%, 23), *Rhizobium* (3.7%, 17), and *Microbacterium* (3.7%, 17). In addition, 193 distinct species were estimated to exist within the collection, with most of the species coming from the genera *Bacillus* (18.7%, 36), followed by *Paenibacillus* (11.9%, 23), *Streptomyces* (11.4%, 22), *Microbacterium* (4.7%, 9), and *Flavobacterium* (3.6%, 7). A greater number of distinct genera were cultured from the root endosphere (49 genera) vs. the rhizosphere (35 genera). Only 16 genera (*Rhodococcus, Microbacterium, Kocuria, Streptomyces, Deinococcus, Bacillus, Lysinibacillus, Paenibacillus, Cohnella, Mesorhizobium, Burkholderia, Achromobacter, Paraburkholderia, Ramlibacter, Massilia*, and *Stenotrophomonas*) were shared between the rhizosphere and root endosphere. For three genera, *Rhizobium, Flavobacterium*, and *Kosakonia*, many isolates were cultured from the root endosphere samples. A few genera (i.e., *Phyllobacterium, Labrys, Ochrobactrum, Skermanella*, and *Cupriavidus*) were cultured only from the rhizosphere, but only a few strains were present within the collection. In addition, various novel species could be isolated, including some members of Alphaproteobacteria, Gammaproteobacteria, and Firmicutes (Supplementary Table S1).

**Table 1:** The list of abbreviations.

**List**	**Abbreviations**
*Ginkgo* root tissue samples	RE
indoor samples using mixed media without any additional carbon sources	MM
indoor samples using mixed media supplemented with starch	GM
indoor samples using mixed media supplemented with glucose	MSM
all enrichment culture samples	ECE
abundance-based coverage	ACE
Fisher's least significant difference	LSD
non-metric multidimensional scaling analysis	NMDS
principal coordinates analysis	PCoA
permutational multivariate analysis of variance	PERMANOVA

### 3.2. Distinct microbiota between the enrichment collections and root tissues

Owing to the possible presence of artificial bias during pure culture, the microbial diversity based on culture-independent methodologies should not be ignored. Bacterial communities of the enrichment collections and root tissues were analyzed using the 16S rRNA gene amplicon sequencing. A total of 2,506,077 high-quality reads from all samples were generated and clustered into 1,597 OTUs. The community richness (Chao1 and ACE), community diversity (Shannon and Simpson), sequencing depth (Observed), and community structure (Fisher) in *Ginkgo* root tissue samples (RE) were significantly different from those in the collection samples. Among them, the Shannon index in RE was significantly higher than that in the collection samples (*P* < 0.05), especially in MSM communities (Figure 1B). In addition, NMDS, PCoA, and PERMANOVA (Figure 1C) of bacterial communities at OTU levels showed that there were significant differences between *in situ* and enriched bacterial taxa (R^2^=0.64; *P* < 0.001).

**Figure 2:**
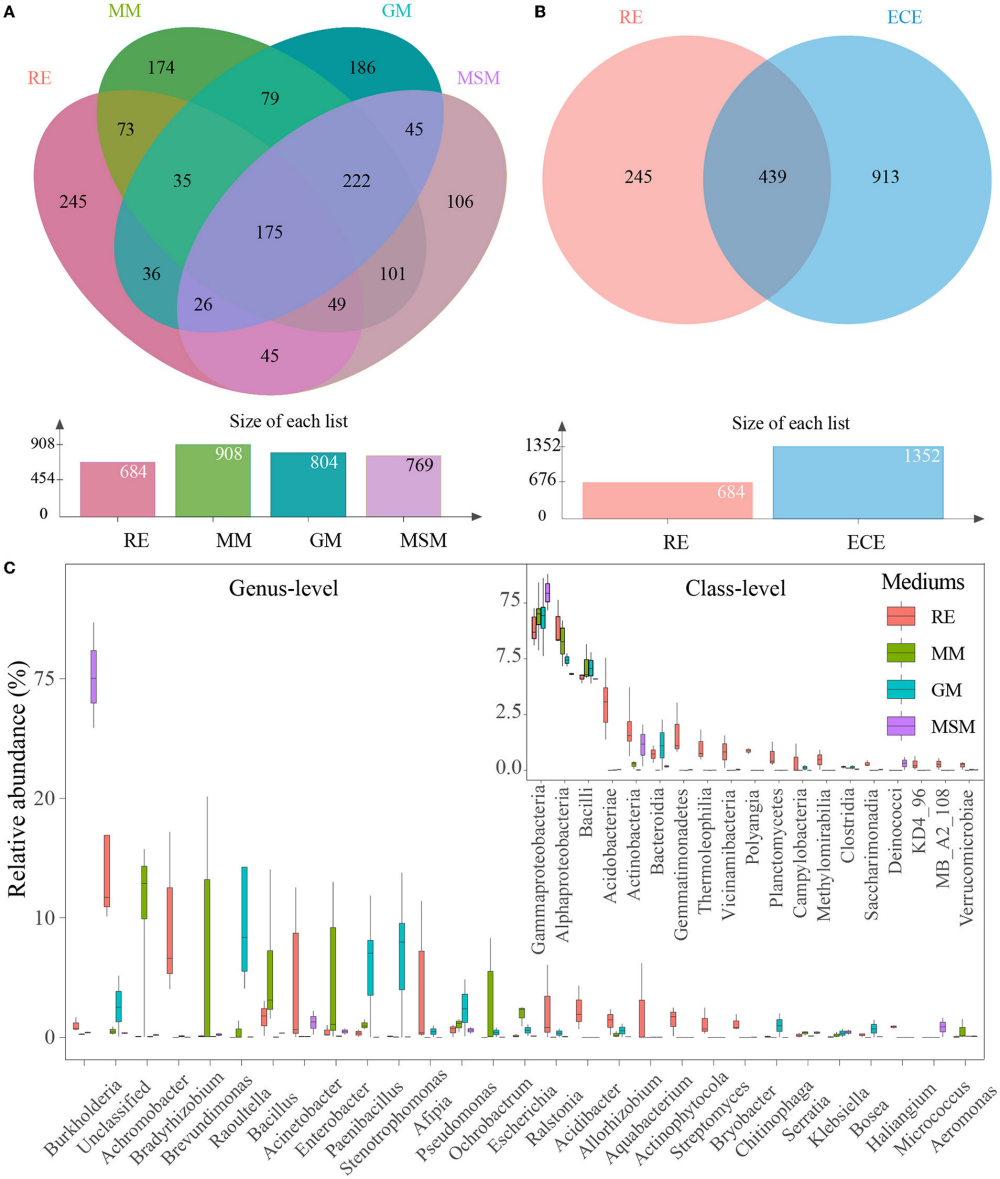
Composition variance of the root endophytic bacterial communities after the enrichment. **(A)** OUTs composition differences between four experimental groups. **(B)** OUT composition differences between the *in situ* root samples and the enrichment collections. **(C)** Composition of shared bacterial taxa (439 OTUs) between the *in situ* root samples and the enrichment collections. These two plots show the top 19 most abundant classes and the top 30 most abundant genera of bacterial communities in all experimental groups, respectively. Table 1 presents a list of abbreviations of the groups used in the figure.

It is interesting to examine differences in the root microbiota of *in situ* samples and enrichment collections at the OTU level. The number of OTUs with 97% similarity varied from 684 to 908 (Figure 2A). The 439 OTUs of bacterial taxa were shared by root samples and enrichment collections, whereas 245 bacterial OTUs were not co-cultured using the mixed media (Figure 2B). The number and relative abundance of shared OTUs accounted for 64.2% and 77.7% of the bacterial taxa in root tissue samples, respectively. However, even for the shared OTUs, their composition and diversity showed a large variation in these enrichment collections (Figure 2A; Supplementary Figure S2). These shared OTUs were classified into the following 19 classes (Figure 2C), mainly including Gammaproteobacteria, Alphaproteobacteria, Bacilli, Actinobacteria, Bacteroidia, Acidobacteriae, and Gemmatimonadetes. The rare or recalcitrant OTUs in root samples were mainly associated with Actinobacteria, Alphaproteobacteria, Blastocatellia, and Ktedonobacteria (Supplementary Figure S3).

To reveal the bacterial taxa dominating inside the *Ginkgo* roots, OTUs with a relative abundance of > 1% were designated as predominant OTUs. A total of 19 predominant OTUs were found, with 16 OTUs common to RE and enrichment collections and 3 OTUs specific to RE, which suggested that roughly 84% of the natural community of root-associated endosphere bacteria could be co-cultured. These OTUs were classified into the following five phyla, including Proteobacteria (9 OTUs), Actinobacteriota (4 OTUs), Acidobacteriota (4 OTUs), Firmicutes (1 OTU), and Gemmatimonadota (1 OTU). We further compared how the core taxa of the interior of the *Ginkgo* roots change after the enrichment. Collectively, a total of 33 core OTUs (i.e., the prevalence in 20% of samples and relative abundance in 0.01%) were predicted in the root samples (Supplementary Figure S4), with 23 OTUs common to the enrichment collections. These OTUs were mainly classified into the following three phyla: Proteobacteria (13 OTUs), Acidobacteriota (6 OTUs), and Actinobacteriota (6 OTUs). Surprisingly, the use of MM, GM, and MSM resulted in the culturing of more than 90.0% (30 out of 33) of the core OTUs in the root samples. Only three OTUs belonging to Acidobacteriota (1 OTU) and Actinobacteriota (2 OTUs) were not cultured using the mixed media. In addition, there are significant differences in the diversity of core OTUs (Supplementary Figure S4) and core families (Supplementary Figure S5) between the root samples and the enrichment collections.

**Figure 3:**
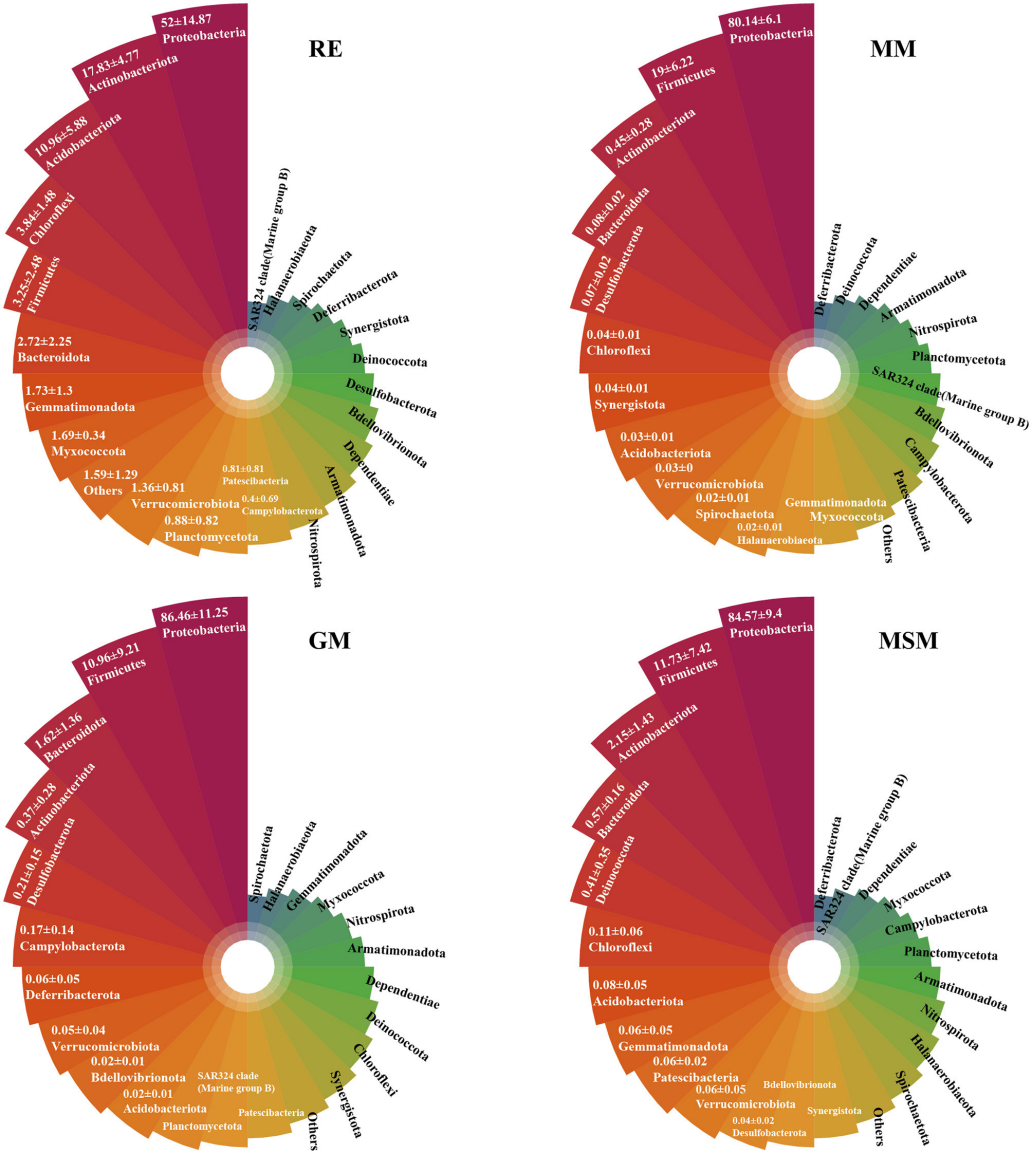
The upper plots show the top 24 most abundant phyla of bacterial communities in the root tissues and the enrichment collections. Table 1 has a list of abbreviations of the groups used in the figure.

A comparison of the bacterial community compositions at the phylum level showed that Proteobacteria (52.0%), Actinobacteriota (17.8%), and Acidobacteriota (11.0%) were the major components of bacterial taxa in the *in situ* root samples, while Proteobacteria and Firmicutes dominated the composition of bacterial taxa across all collection samples (Figure 3; Supplementary Figure S6). Intriguingly, some OTUs belonging to Crenarchaeota and Euryarchaeota are present in MSM. The phyla, such as Caldatribacteriota (Fonseca et al., 2022), which generally live in the anoxic layers were also co-cultured using these three media, such as MM, MSM, and GM (Supplementary Figure S6). In contrast, some phyla, such as Zixibacteria, displayed an unculturable trait. At the family level (Supplementary Figure S7), the best-represented bacterial families in the *in situ* root samples mainly included *Xanthobacteraceae* (26.3%), *Moraxellaceae* (9.8%), *Acidothermaceae* (7.7%), and *Burkholderiaceae* (5.7%). The five most abundant families in MM were *Yersiniaceae* (32.7%), followed by *Alcaligenaceae* (16.1%), *Caulobacteraceae* (13.0%), *Enterobacteriaceae* (10.9%), and *Bacillaceae* (9.4%). Through the supplements of glucose and starch, *Yersiniaceae* (54.8%) and *Burkholderiaceae* (81.1%) became dominant in GM and MSM, respectively.

### 3.3. OTUs enrichment under indoor conditions

**Figure 4:**
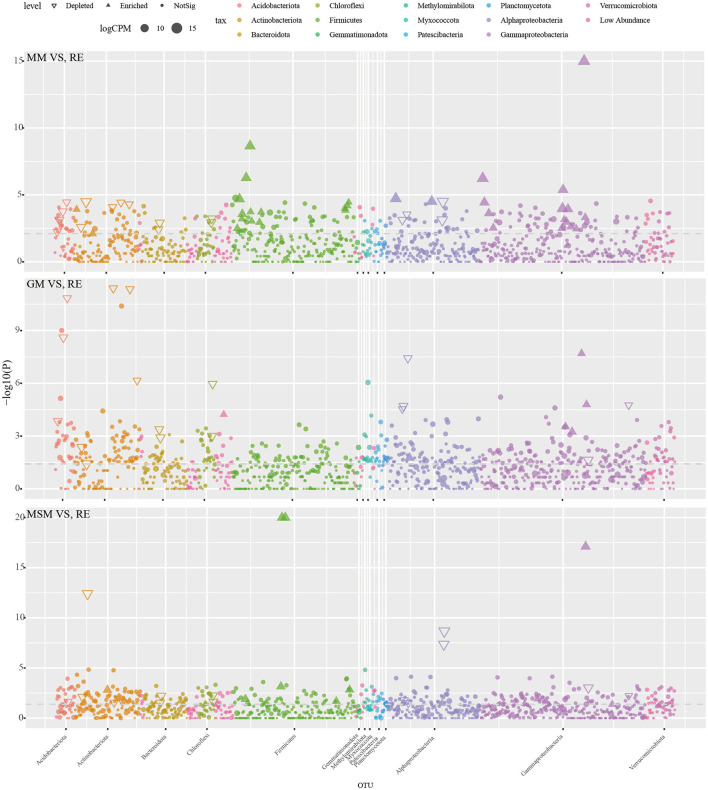
The Manhattan plot illustrating the enrichment and depletion patterns of bacterial microbiota in the enrichment collections compared with the *in situ* root tissues. Table 1 presents a list of abbreviations of the groups used in the figure.

It should be noted that most of the bacterial taxa in the root endosphere were not significantly enriched using those mixed media. Manhattan plots (Figure 4; Supplementary Table S2) were used to analyze the enrichment of OTUs on the basis of their taxonomic category. Among the enrichment collections, the depleted OTUs (20.3% in the root endosphere) were associated with a wide range of bacterial phyla, namely, Acidobacteria, Actinobacteria, Bacteroidetes, Proteobacteria, and Chloroflexi (FDR-adjusted *P* < 0.05, Wilcoxon's rank-sum test). By contrast, 32 OTUs (0.6% in the root endosphere) significantly enriched in MM mainly belonged to Proteobacteria and Firmicutes (FDR-adjusted *P* < 0.05, Wilcoxon's rank-sum test). With the supplement of carbon sources, GM retained the capacity to significantly enrich 6 OTUs (< 0.01% in the root endosphere) belonging to Proteobacteria and Elusimicrobiota (FDR-adjusted *P* < 0.05), while MSM retained the capacity to significantly enrich 19 OTUs (0.2% in the root endosphere) belonging to Proteobacteria, Firmicutes, Actinobacteriota, Bacteroidota, Gemmatimonadota, and Desulfobacteriota.

### 3.4. Discrepant metabolic profiles

**Figure 5:**
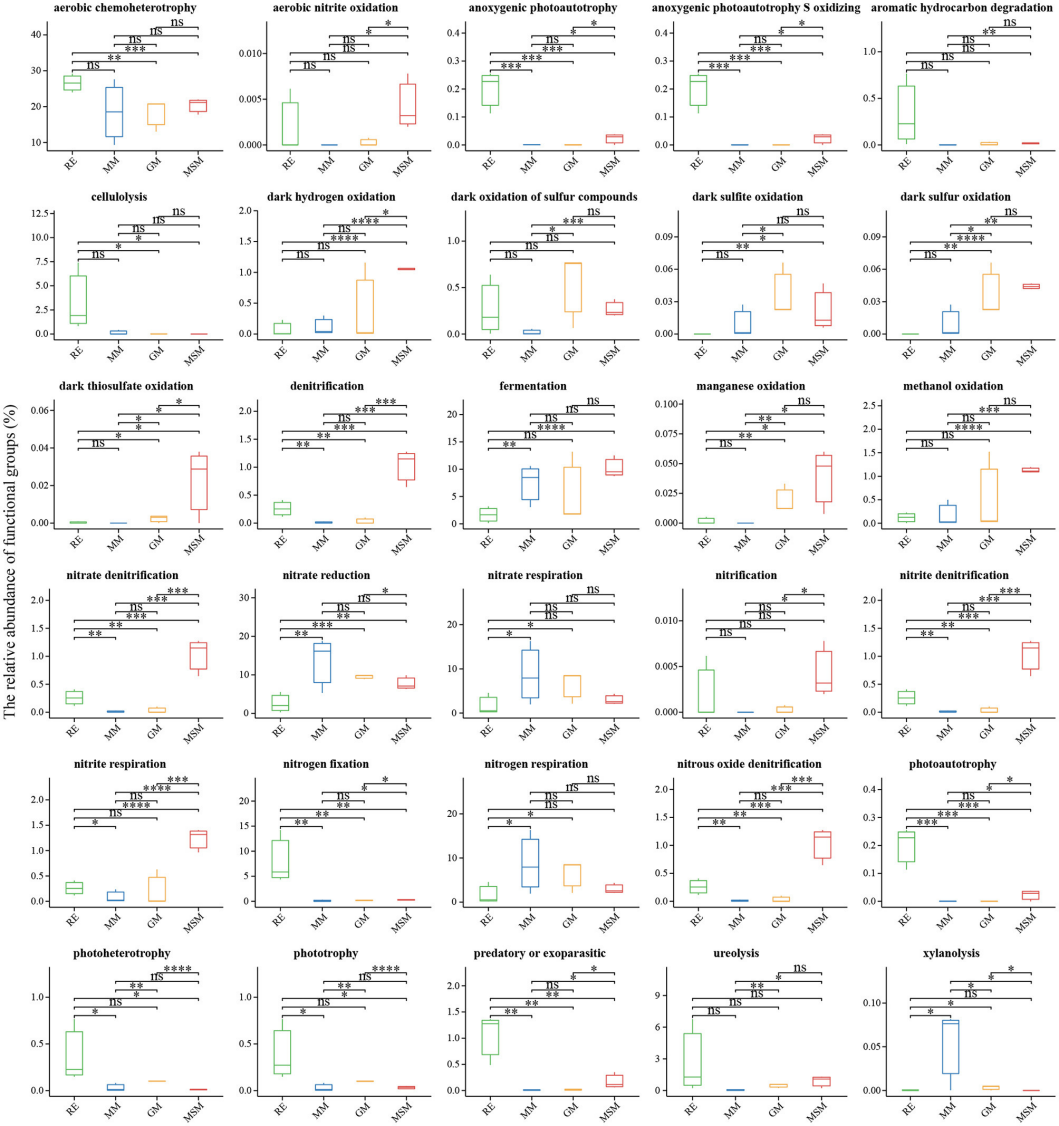
Functional prediction of the bacterial communities in all experimental groups using FAPROTAX. The results revealed the significant differences in the potential functions of the bacterial communities between the *in situ* samples and the enrichment collections. ^*^Correlation is significant at the 0.05 level; ^**^Correlation is significant at the 0.01 level; ^***^Correlation is significant at the 0.001 level; ^****^Correlation is significant at the 0.0001 level. Table 1 presents a list of abbreviations of the groups used in the figure.

Using the FAPROTAX classifier, we found that the 398 OTUs (24.9% of the total OTUs) were mainly assigned to 55 pathways represented by 30 significant variational functions (Figure 5). The domain function was linked to chemoheterotrophy. Unlike the enrichment collections, the endophytic bacterial communities from RE have many functions associated with various factors such as aerobic chemoheterotrophy, photoautotrophy, aromatic hydrocarbon degradation, cellulolysis, nitrogen fixation, photoheterotrophy, phototrophy, and ureolysis. Compared with RE, functions associated with the sulfur cycle were solely enriched in these three media, such as MM, MSM, and GM, such as sulfur respiration, dark sulfur oxidation, sulfite respiration, sulfate respiration, dark sulfite oxidation, respiration of sulfur compounds, and thiosulfate respiration. For instance, in addition to the sulfur metabolism, MSM significantly increased the relative abundance of photoautotrophy, dark hydrogen oxidation, and many nitrogen metabolisms, including aerobic nitrite oxidation, denitrification, nitrous oxide denitrification, and nitrite respiration (*p* < 0.05). Notably, the cumulative abundance of nitrogen-related OTUs enriched in the enrichment collections (>100 OTUs) was superior to that of sulfur-related OTUs (~15 OTUs). Based on the prediction of Tax4Fun2, both MSM and RE harbored a significantly higher relative abundance of metabolism (e.g., substrate dependence, xenobiotic biodegradation and metabolism, and the metabolism of terpenoids and polyketides) than GM and MM (Supplementary Figure S8; *p* < 0.05). Compared to MSM and RE, MM and GM significantly increased the relative abundance of environmental information processing and genetic information processing (e.g., translation, replication, and repair).

### 3.5. Co-occurrence network structure of bacterial OTUs

**Figure 6:**
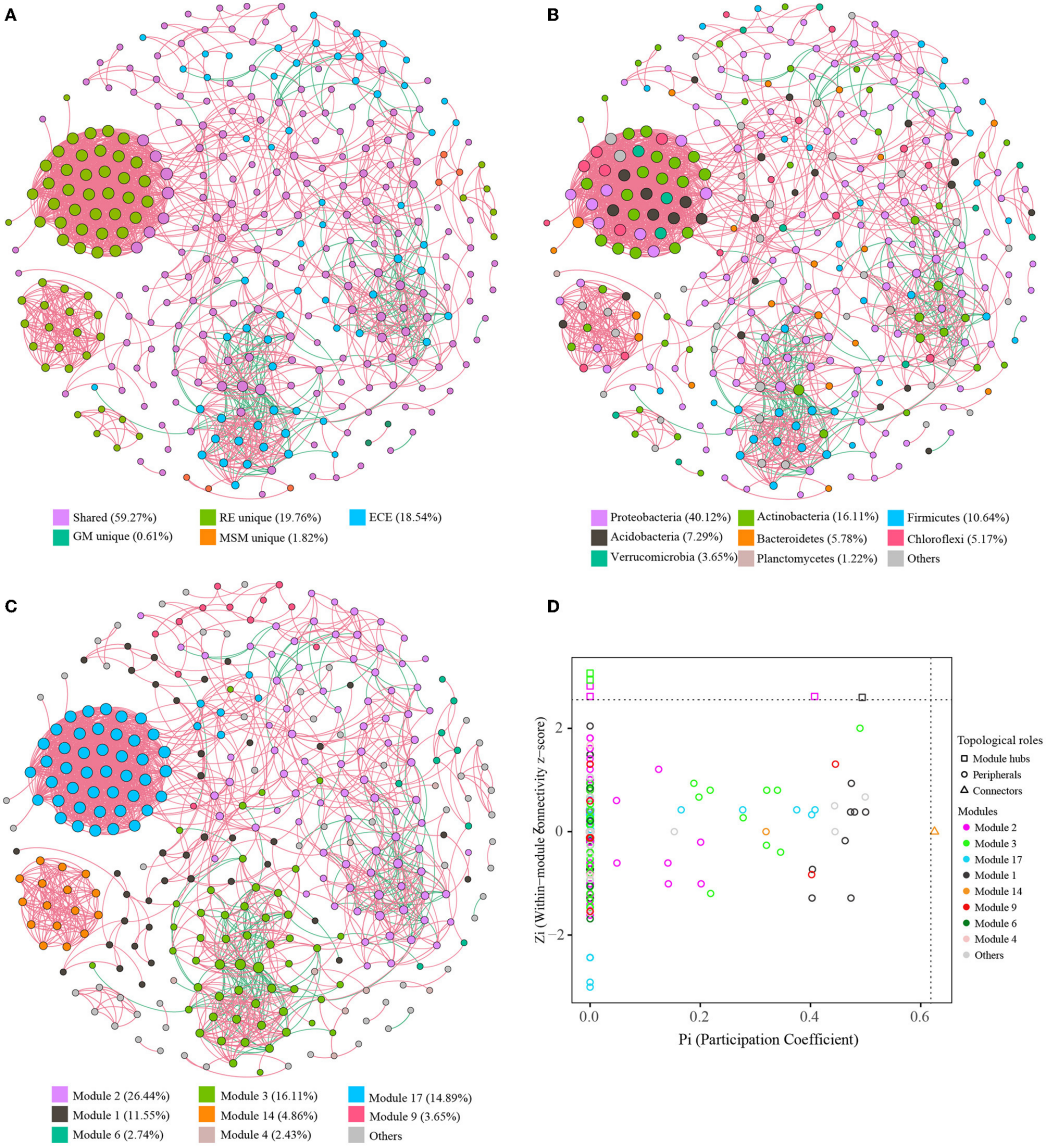
Characteristics of co-occurrence networks with OTUs. Co-occurrence networks with OTUs colored by the grouping **(A)**, taxonomy **(B)**, and modularity **(C)**, successively. Nodes represent the retained OTUs under the strict threshold. Edges (red lines) represent statistically significant positive correlations of each OTU pair, while edges (blue lines) represent statistically significant negative correlations of each OTU pair. The size of nodes represents the connective degree of each node in the network. **(D)** The distribution of bacterial OTUs on the Zi-*Pi* plot based on their topological characteristics. Table 1 presents a list of abbreviations for the groups used in the figure.

The co-occurrence patterns of bacterial endophytes in *Ginkgo* roots and the enrichment collections were analyzed using the interspecies and interfamily topological network analysis (Figure 6; Supplementary Figure S9). Spearman's network for all samples had 329 nodes connected by 3,836 edges with 0.10 of degree centralization, classifying eight dominant modules (>2% of total nodes; Figure 6). We observed that 329 OTUs represented roughly 58.6% of bacterial community compositions in each sample on average. Among different groups, roughly 80.8%, 52.6%, 87.3%, and 13.7% of bacterial community compositions in RE, MM, GM, and MSM samples were involved in the network (Figure 6), respectively. Unique OTUs affiliated with RE clustered into four subnetworks and were mainly dominant in module 17 and module 14. The proportion of negative edges was 10.6% and most of them were located in modules 2 and 3. The core nodes in the network were Proteobacteria, Actinobacteria, Firmicutes, Acidobacteria, Bacteroidetes, Verrucomicrobia, Chloroflexi, and Planctomycetes. Importantly, there were 7 shared OTUs in module 17 performing a high connective degree, which linked the natural community to the enrichment collections. Based on Zi and Pi values for the network, most of the nodes were classified as peripherals and six nodes belonging to Proteobacteria and Actinobacteria were the module hubs (Figure 6D). In addition, the SparCC network (Supplementary Figure S9) constructed within MicrobiomeAnalyst showed more significant correlations among families dominated by the enrichment samples than *in situ* root tissues, especially for the experimental groups such as GM and MSM.

## 4. Discussion

Microbial communities associated with medicinal plants are being considered as a lever to promote the synthesis of plant secondary metabolites. Especially, endophytic bacteria take part in the secondary metabolic processes of plant trees and contribute to plant growth, nutrient uptake, and systemic resistance (Daffonchio et al., 2015; Zou et al., 2021). While high-throughput sequencing can provide us insights into the structure and functional potential of microbial community, the diversity of bacterial endophytes is highly underestimated. To capture as much diversity as possible, we have reported 455 unique bacterial isolates (Supplementary Table S1) and explored the cultivable potential of the *in situ* bacterial endophytes using simply modified media (a mixed medium without any additional carbon sources [MM] and two other mixed media with separately added starch [GM] and supplemented glucose [MSM]). These bacterial isolates derive from the root endosphere and rhizosphere and represent 8 classes, 20 orders, 42 families, and 67 genera from five phyla: Actinobacteria, Bacteroidetes, Firmicutes, Proteobacteria, and Deinococcus-Thermus. Additionally, the enrichment collections include a large fraction of the natural bacterial community, as assessed by a comparison to amplicon data. In addition, we demonstrated that the enrichment was necessary and useful to assess bacterial diversity and interspecies interaction.

As reported in a previous study (Rodriguez et al., 2019; Fadiji et al., 2020; Ghosh et al., 2021), many bacterial isolates (i.e., *Bacillus, Paenibacillus, Rhizobium, Pseudomonas, Streptomyces, Sphingobium, Burkholderia*, and *Chitinophaga*) within the culture collections may have plant growth-promoting attributes and interact with complex eukaryotes. These plant growth-promoting strains are well-known for nitrogen fixation, solubilizing phosphorus, siderophore production, phytohormone production, antibiotics production, or systemic resistance. In addition, we further observed that various novel species were collected when using these modified media, including Alphaproteobacteria, Gammaproteobacteria, and Firmicutes (Supplementary Table S1), which further indicated that the culture work is effective to capture microbial diversity. However, while many functionally valuable microorganisms with enormous quantities of biomass under common culture conditions were obtained, it was not comfortable to use this enrichment method mentioned in the present study to isolate some recalcitrant bacteria, such as the rarely cultivated phyla such as Verrucomicrobiota and Acidobacteriota. Even so, this method can be improved using some simply modified methods to prepare the media, such as the use of gellan gum as a gelling reagent (Nishioka and Tamaki, 2022). Additionally, the related results showed using one medium to culture and isolate will limit the findings of bacterial diversity, but diversified media may capture certain community diversity and metabolic function.

Owing to the presence of artificial bias during pure culture, the microbial diversity based on culture-independent methodologies should not be ignored. Proteobacteria, Actinobacteriota, and Acidobacteriota dominated the composition of *in situ* endophytic bacteria, consistent with a previous study (Shehata et al., 2021), while the bacterial taxa of the enrichment were dominated by Proteobacteria and Firmicutes. Notably, roughly 77% of the natural community of root-associated endophytes were predicted to have successfully cultivated the possibility based on a comparison of the 16S rRNA gene sequences between the enrichment collections and the *Ginkgo* root endophyte community. In addition, the use of MM, GM, and MSM may be effective to culture more than 80% (16 out of 19) of the predominant OTUs and over 90% (30 out of 33) of the core OTUs in the root samples. These results indicated that a certain percentage of the rare or recalcitrant taxa in the root endosphere was difficult to be cultured in the indoor enrichment using the existing media, which mainly include Actinobacteria, Alphaproteobacteria, Blastocatellia, and Ktedonobacteria. Especially, the enriched results showed that 20.3% OTUs in the root endosphere were depleted after the enrichment cultivation, which were associated with Acidobacteria, Actinobacteria, Bacteroidetes, Proteobacteria, and Chloroflexi. By contrast, some OTUs were significantly enriched after the enrichment cultivation. Among them, more OTUs (0.6% in the root endosphere) were significantly enriched in MM than in GM and MSM. This indicated that the supplement of carbon sources intensified the competition among microorganisms within the community. Similar to a previous study on marine sediment (Mu et al., 2018), several novel distinct OTUs appeared after the enrichment cultivation because sequencing analyses could miss some low-abundant sequences of rare strains, such as the real microbial dark matter in nature samples. However, the proportion of novel OTUs was obviously greater after the enrichment treatment in the plant root samples than the marine sediment. The fact is that the enrichment culture could change some low-abundant OTUs to become abundant and reach the detection threshold (Mu et al., 2018; Carper et al., 2021). These results strongly indicate that the *Gingko* root endosphere contains a set of rare or recalcitrant taxa that can closely interact with their host plant and barely survive until the proper nutrients arrive (Metcalf et al., 2016).

Large differences in microbial functions between the root endosphere and the enrichment collection are displayed. The results of the function prediction showed that the bacterial taxa of the root endosphere had strong metabolisms with the representative of aerobic chemoheterotrophy, while the functions of the enrichment collections were represented by sulfur metabolism. The *in situ* metabolic profile of the root endosphere is difficult to replicate in a laboratory. Even microbes of the same species can show different metabolisms when glucose is the major carbon (Kimes et al., 2014). Thus, the exploration of the endophytic microbial function needs to be carried out from multiple aspects. On the one hand, we can learn the metabolic profile of plant microbiota through an individual strain. For instance, using the genomic information on several isolates, researchers have found that the genomes of plant-associated bacteria encode more genes associated with carbohydrate metabolism and fewer mobile elements compared with related non-plant-associated genomes (Levy et al., 2017). In addition, two sets of plant-associated genes about plant colonization and microbe–microbe competition were identified (Levy et al., 2017). This can be helpful to study the endophytic microbial function and provide theoretical guidance for the improvement of enrichment culture methods. On the other hand, at the community level, multi-omics methods have been used to explore the community function in the root endosphere combined with the enrichment culture. As previously reported, the functions of bacterial endophytes do not rely on taxonomy groupings but are affected by the environmental factors and the plant host (Burke et al., 2011; Hardoim et al., 2015). We can use the substrate supplement to strengthen enrichment systems to impact microbial functions and explore the rare functions. Our results further suggest that the exploration of the endophytic microbial function requires constant integration and comparison.

Furthermore, based on these simple enough communities provided by mixed cultures, we can completely understand the communication of microbes and assess the potential relationships between microbes. Notably, unique OTUs in the root endosphere were clustered into four closely related subnetworks, which were composed of those unsuccessfully cultivated taxa. Microbial hub species identified through an interspecies network analysis could stand for generalist microorganisms that are abundantly and reproducibly involved in plant bodies (Hassani et al., 2018). Even so, the shared OTUs closely related to unique taxa within the root endosphere are more important and helpful to grow recalcitrant bacteria. Especially, there were 7 OTUs in module 17 performing high connective degree, which linked the natural community to the enrichment collections. If we attempt to enrich their abundance in indoor flasks, maybe more uncultured taxa within the root endosphere can become available to be isolated. Particularly, the fact is that almost all of the negative edges were located in the enrichment collection. This feature can be used as a pre-filtering approach for the selection of potential antagonists as part of biocontrol strategies based on the negative relationships (Cobo-Díaz et al., 2019). Then, more studies concerning culture-dependent approaches should be done to verify these potential antagonists, both in laboratory and in field experiments. This network analysis can provide a foundation for further exploration into the community assembly and function of the *Ginkgo* root endosphere.

**Figure 7:**
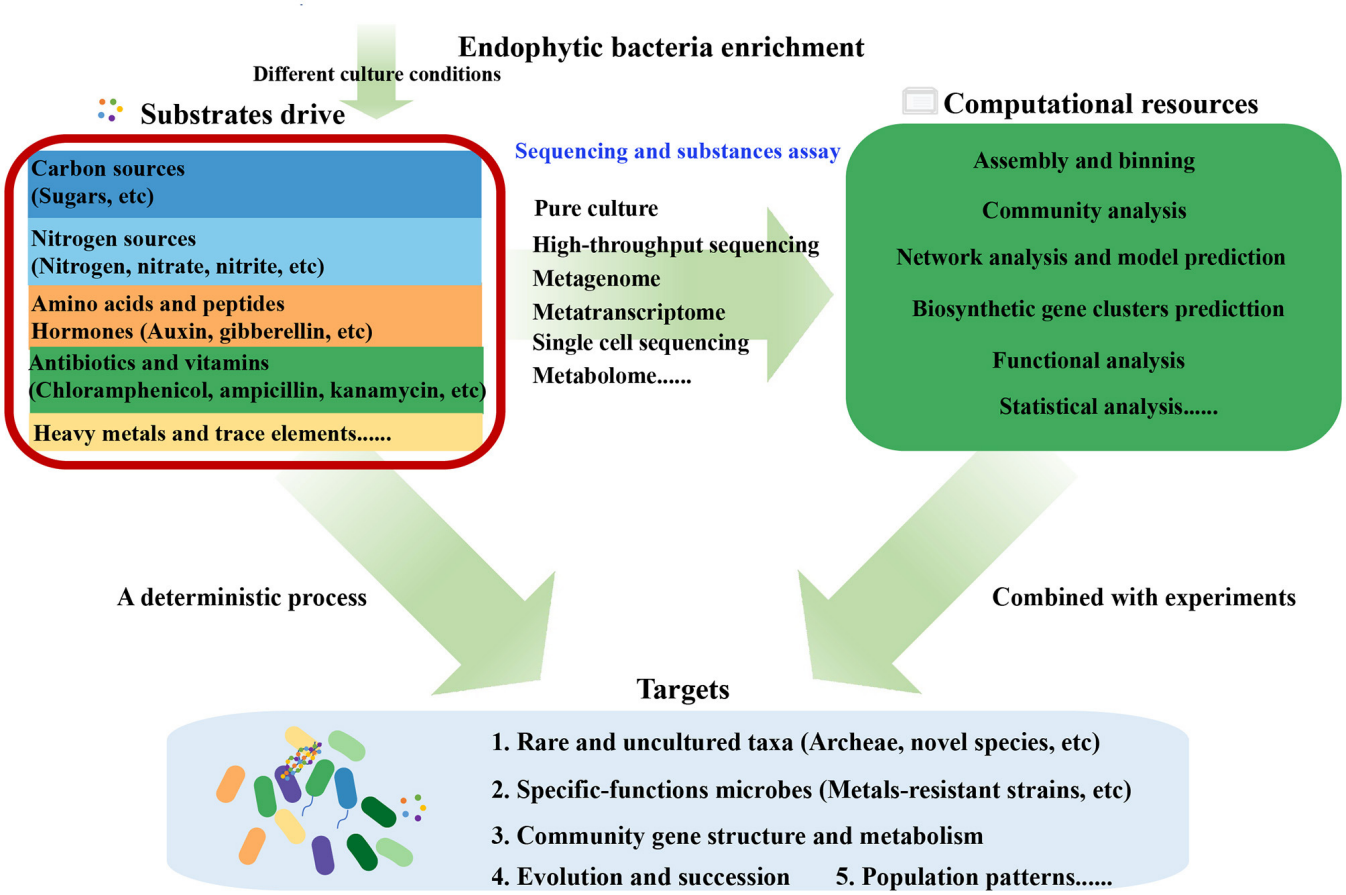
The outline of various substrate-based approaches to investigate the composition, function, correlation, and metabolism of plant endophytic microbial communities.

Microbes can be co-cultured in a laboratory setting, which suggests the possibility of obtaining a pure culture. Enrichment in a laboratory can be the first step to culture endophytic bacteria (Figure 7). Enrichment for specific taxa or species of microbes can only be achieved by defining conditions that will preferentially allow them to grow. For example, a complicated mixed culture system was constructed to obtain high-efficiency isolation of uncultured strains from the marine sediment, which contained the competition, cooperation, or coordination among bacterial communities (Mu et al., 2018). In the future, the microbial exploration of plant endosphere environments should stay in the spotlight, not only as cultivation-dependent studies but also as studies focused on community construction and plant–microbe co-culture. Cultivation-dependent studies mainly include culturomics and cultivation improvements (Kapinusova et al., 2022), which are necessary to largely obtain microorganisms that have specific functions. On the one hand, the collected microorganisms can be used to elucidate their ecological roles and biosynthesize their own secondary metabolites. On the other hand, these microorganisms can be selected and constructed a community, which is useful to explore the plant–microbe interaction and promote the biosynthesis of plant's secondary metabolites (Carper et al., 2021; Li et al., 2023). Thus, the future of endophytic microbes is as bright as their demand for their utilization in agriculture and medicine as well as in industry, which will enlarge day by day.

## 5. Conclusion

In the present study, 455 bacterial isolates cultured from the *Ginkgo* root endosphere and rhizosphere were collected. A series of plant growth-promoting endophytes had multiple representatives within the culture collection. In addition to the ecological significance, these functionally valuable microorganisms with enormous quantities of biomass under common culture conditions can be used to promote the production of plant potential secondary metabolites. The amplicon analysis indicated that a large fraction of the natural community of root-associated endophytes was successfully presented in the enrichment collection. We further found that the bacterial taxa of the root endosphere had strong metabolisms with the representative of aerobic chemoheterotrophy, while the functions of the enrichment collections were represented by sulfur metabolism. In addition, the interspecies network analysis suggested that the substrate supplement could significantly impact bacterial interactions within the enrichment collections. The results supported that it is better to use the enrichment culture to assess the cultivable potential and the interspecies interaction as well as to increase the detection/isolation of certain bacterial taxa. Overall, this study will deepen our knowledge of the indoor endophytic culture and provide important insights into substrate-driven enrichment.

## Data availability statement

The datasets presented in this study can be found in online repositories. The names of the repository/repositories and accession number(s) can be found below: https://www.ncbi.nlm.nih.gov/, PRJNA889250.

## Author contributions

SZ analyzed the data and wrote the manuscript. CS assisted in analyzing some data and recertifying the results. XL and YL revised the manuscript. All authors read and approved the submitted manuscript.

## References

[B1] Attia S. Russel J. Mortensen M. S. Madsen J. S. Sørensen S. J. (2022). Unexpected diversity among small-scale sample replicates of defined plant root compartments. ISME J. 16, 997–1003. 10.1038/s41396-021-01094-7 34759302 PMC8940884

[B2] Beckers B. Op De Beeck M. Weyens N. Boerjan W. Vangronsveld J. (2017). Structural variability and niche differentiation in the rhizosphere and endosphere bacterial microbiome of field-grown poplar trees. Microbiome 5, 25. 10.1186/s40168-017-0241-2 28231859 PMC5324219

[B3] Bolyen E. Rideout J. R. Dillon M. R. Bokulich N. A. Abnet C. C. Al-Ghalith G. A.. (2019). Reproducible, interactive, scalable and extensible microbiome data science using QIIME 2. Nat. Biotechnol. 37, 852–857. 10.1038/s41587-019-0209-9 31341288 PMC7015180

[B4] Bulgarelli D. Schlaeppi K. Spaepen S. Van Themaat E. V. L. Schulze-Lefert P. (2013). Structure and functions of the bacterial microbiota of plants. Annu. Rev. Plant Biol. 64, 807–838. 10.1146/annurev-arplant-050312-120106 23373698

[B5] Burke C. Steinberg P. Rusch D. Kjelleberg S. Thomas T. (2011). Bacterial community assembly based on functional genes rather than species. Proc. Natl Acad. Sci. USA 108, 14288–14293. 10.1073/pnas.1101591108 21825123 PMC3161577

[B6] Carper D. L. Weston D. J. Barde A. Timm C. M. Lu T.-Y. Burdick L. H.. (2021). Cultivating the bacterial microbiota of *Populus* roots. Msystems 6, e01306–01320. 10.1128/mSystems.01306-20 34156297 PMC8269261

[B7] Chen L. Fang K. Zhou J. Yang Z.-P. Dong X.-F. Dai G.-H.. (2019). Enrichment of soil rare bacteria in root by an invasive plant Ageratina adenophora. Sci. Total Environ. 683, 202–209. 10.1016/j.scitotenv.2019.05.220 31132698

[B8] Chong J. Liu P. Zhou G. Xia J. (2020). Using MicrobiomeAnalyst for comprehensive statistical, functional, and meta-analysis of microbiome data. Nat. Protoc. 15, 799–821. 10.1038/s41596-019-0264-1 31942082

[B9] Cobo-Díaz J. F. Baroncelli R. Le Floch G. Picot A. (2019). Combined metabarcoding and co-occurrence network analysis to profile the bacterial, fungal and fusarium communities and their interactions in maize stalks. Front. Microbiol. 10, 261. 10.3389/fmicb.2019.00261 30833940 PMC6387940

[B10] Compant S. Cambon M. C. Vacher C. Mitter B. Samad A. Sessitsch A. (2021). The plant endosphere world—bacterial life within plants. Environ. Microbiol. 23, 1812–1829. 10.1111/1462-2920.15240 32955144

[B11] Compant S. Clément C. Sessitsch A. (2010). Plant growth-promoting bacteria in the rhizo-and endosphere of plants: their role, colonization, mechanisms involved and prospects for utilization. Soil Biol. Biochem. 42, 669–678. 10.1016/j.soilbio.2009.11.024

[B12] Daffonchio D. Hirt H. Berg G. (2015). “Plant-microbe interactions and water management in arid and saline soils,” in Principles of Plant-Microbe Interactions. (Springer) 265–276. 10.1007/978-3-319-08575-3_28

[B13] Dwibedi V. Rath S. K. Joshi M. Kaur R. Kaur G. Singh D.. (2022). Microbial endophytes: application towards sustainable agriculture and food security. Appl. Microbiol. Biotechnol. 106, 5359–5384. 10.1007/s00253-022-12078-8 35902410

[B14] Edgar R. C. (2010). Search and clustering orders of magnitude faster than BLAST. Bioinformatics 26, 2460–2461. 10.1093/bioinformatics/btq461 20709691

[B15] Edgar R. C. (2013). UPARSE: highly accurate OTU sequences from microbial amplicon reads. Nat. Methods 10, 996–998. 10.1038/nmeth.2604 23955772

[B16] Fadiji A. E. Ayangbenro A. S. Babalola O. O. (2020). Metagenomic profiling of the community structure, diversity, and nutrient pathways of bacterial endophytes in maize plant. Antonie van Leeuwenhoek 113, 1559–1571. 10.1007/s10482-020-01463-w 32803452

[B17] Fitzpatrick C. R. Salas-Gonzalez I. Conway J. M. Finkel O. M. Gilbert S. Russ D.. (2020). The plant microbiome: from ecology to reductionism and beyond. Annu. Rev. Microbiol. 74, 81–100. 10.1146/annurev-micro-022620-014327 32530732

[B18] Fonseca A. Espinoza C. Nielsen L. P. Marshall I. P. G. Gallardo V. A. (2022). Bacterial community of sediments under the Eastern boundary current system shows high microdiversity and a latitudinal spatial pattern. Front. Microbiol. 13, 1016418. 10.3389/fmicb.2022.1016418 36246233 PMC9561620

[B19] Friedman J. Alm E. J. (2012). Inferring correlation networks from genomic survey data. PLoS Comput. Biol. 8, e1002687. 10.1371/journal.pcbi.1002687 23028285 PMC3447976

[B20] Ghosh S. Bhagwat T. Webster T. J. (2021). “Endophytic Microbiomes and Their Plant Growth-Promoting Attributes for Plant Health,” in Current Trends in Microbial Biotechnology for Sustainable Agriculture, eds. A. N. Yadav, J. Singh, C. Singh and N. Yadav. (Singapore: Springer Singapore) 245–278. 10.1007/978-981-15-6949-4_11

[B21] Gottel N. R. Castro H. F. Kerley M. Yang Z. Pelletier D. A. Podar M.. (2011). Distinct microbial communities within the endosphere and rhizosphere of *Populus deltoides* roots across contrasting soil types. Appl. Environ. Microbiol. 77, 5934–5944. 10.1128/AEM.05255-11 21764952 PMC3165402

[B22] Hardoim P. R. Van Overbeek L. S. Berg G. Pirttil,ä A. M. Compant S. Campisano A.. (2015). The hidden world within plants: ecological and evolutionary considerations for defining functioning of microbial endophytes. Microbiol. Mol. Biol. Rev. 79, 293–320. 10.1128/MMBR.00050-14 26136581 PMC4488371

[B23] Hassani M. A. Duran P. Hacquard S. (2018). Microbial interactions within the plant holobiont. Microbiome 6, 58. 10.1186/s40168-018-0445-0 29587885 PMC5870681

[B24] Hiraoka S. Hirai M. Matsui Y. Makabe A. Minegishi H. Tsuda M.. (2020). Microbial community and geochemical analyses of trans-trench sediments for understanding the roles of hadal environments. ISME J. 14, 740–756. 10.1038/s41396-019-0564-z 31827245 PMC7031335

[B25] Hug L. A. Baker B. J. Anantharaman K. Brown C. T. Probst A. J. Castelle C. J.. (2016). A new view of the tree of life. Nat. Microbiol. 1, 16048. 10.1038/nmicrobiol.2016.48 27572647

[B26] Ikeda S. Kaneko T. Okubo T. Rallos L. E. Eda S. Mitsui H.. (2009). Development of a bacterial cell enrichment method and its application to the community analysis in soybean stems. Microb. Ecol. 58, 703–714. 10.1007/s00248-009-9566-0 19662454

[B27] Jiao J. Y. Wang H. X. Zeng Y. Shen Y. M. (2006). Enrichment for microbes living in association with plant tissues. J. Appl. Microbiol. 100, 830–837. 10.1111/j.1365-2672.2006.02830.x 16553739

[B28] Kaeberlein T. Lewis K. Epstein S. S. (2002). Isolating “Uncultivable” microorganisms in pure culture in a simulated matural environment. Science 296, 1127–1129. 10.1126/science.1070633 12004133

[B29] Kapinusova G. Jani K. Smrhova T. Pajer P. Jarosova I. Suman J.. (2022). Culturomics of bacteria from radon-saturated water of the world's oldest radium mine. Microbiol. Spectr. 10, e01995–e01922. 10.1128/spectrum.01995-22 36000901 PMC9602452

[B30] Kimes N. E. López-Pérez M. Ausó E. Ghai R. Rodriguez-Valera F. (2014). RNA sequencing provides evidence for functional variability between naturally co-existing *Alteromonas macleodii* lineages. BMC Genom. 15, 938. 10.1186/1471-2164-15-938 25344729 PMC4223743

[B31] Leff J. W. Del Tredici P. Friedman W. E. Fierer N. (2015). Spatial structuring of bacterial communities within individual *Ginkgo biloba* trees. Environ. Microbiol. 17, 2352–2361. 10.1111/1462-2920.12695 25367625

[B32] Levy A. Salas Gonzalez I. Mittelviefhaus M. Clingenpeel S. Herrera Paredes S. Miao J.. (2017). Genomic features of bacterial adaptation to plants. Nat. Genet. 50, 138–150. 10.1038/s41588-017-0012-9 29255260 PMC5957079

[B33] Li Z. Xiong K. Wen W. Li L. Xu D. (2023). Functional endophytes regulating plant secondary metabolism: current status, prospects and applications. Int. J. Mol. Sci. 24, 1153. 10.3390/ijms24021153 36674663 PMC9867233

[B34] Lodewyckx C. Vangronsveld J. Porteous F. Moore E. R. Taghavi S. Mezgeay M.. (2002). Endophytic bacteria and their potential applications. Crit. Rev. Plant Sci. 21, 583–606. 10.1080/0735-260291044377

[B35] Louca S. Parfrey L. W. Doebeli M. (2016). Decoupling function and taxonomy in the global ocean microbiome. Science 353, 1272–1277. 10.1126/science.aaf4507 27634532

[B36] Metcalf J. L. Xu Z. Z. Weiss S. Lax S. Van Treuren W. Hyde E. R.. (2016). Microbial community assembly and metabolic function during mammalian corpse decomposition. Science 351, 158–162. 10.1126/science.aad2646 26657285

[B37] Miliute I. Buzaite O. Baniulis D. Stanys V. (2015). Bacterial endophytes in agricultural crops and their role in stress tolerance: a review. Zemdirbyste 102, 465–478. 10.13080/z-a.2015.102.060

[B38] Mu D. S. Du Z. J. Chen J. Austin B. Zhang X. H. (2021). What do we mean by viability in terms of ‘viable but non-culturable' cells? Env. Microbiol. Rep. 13, 248–252. 10.1111/1758-2229.12953 33900036

[B39] Mu D. S. Liang Q. Y. Wang X. M. Lu D. C. Shi M. J. Chen G. J.. (2018). Metatranscriptomic and comparative genomic insights into resuscitation mechanisms during enrichment culturing. Microbiome 6, 1–15. 10.1186/s40168-018-0613-2 30587241 PMC6307301

[B40] Murray M. Thompson W. (1980). Rapid isolation of high molecular weight plant DNA. Nuc. Acids Res. 8, 4321–4326. 10.1093/nar/8.19.4321 7433111 PMC324241

[B41] Nai C. Meyer V. (2018). From axenic to mixed cultures: Technological advances accelerating a paradigm shift in microbiology. Trends Microbiol. 26, 538–554. 10.1016/j.tim.2017.11.004 29191399

[B42] Nishioka T. Tamaki H. (2022). Improved cultivation and isolation of diverse endophytic bacteria inhabiting dendrobium roots by using simply modified agar media. Microbiol. Spectr. 5, e02238–e02222. 10.1128/spectrum.02238-22 36301116 PMC9769524

[B43] Papik J. Folkmanova M. Polivkova-Majorova M. Suman J. Uhlik O. (2020). The invisible life inside plants: deciphering the riddles of endophytic bacterial diversity. Biotechnol. Adv. 44, 107614. 10.1016/j.biotechadv.2020.107614 32858117

[B44] Pundir R. K. Rana S. Kaur A. Kashyap N. Jain P. (2014). Bioprospecting potential of endophytic bacteria isolated from indigenous plants of Ambala (Haryana, India). Int. J. Pharm. Sci. Res. 5, 2309. 10.13040/IJPSR.0975-8232.5

[B45] Quast C. Pruesse E. Yilmaz P. Gerken J. Schweer T. Yarza P.. (2013). The SILVA ribosomal RNA gene database project: improved data processing and web-based tools. Nucleic Acids Res. 41, D590–D596. 10.1093/nar/gks1219 23193283 PMC3531112

[B46] Raaijmakers J. M. Kiers E. T. (2022). Rewilding plant microbiomes. Science 378, 599–600. 10.1126/science.abn6350 36356130

[B47] Raina J.-B. Tapiolas D. Willis Bette L. Bourne David G. (2009). Coral-associated bacteria and their role in the biogeochemical cycling of sulfur. Appl. Environ. Microbiol. 75, 3492–3501. 10.1128/AEM.02567-08 19346350 PMC2687302

[B48] Reinhold-Hurek B. Hurek T. (2011). Living inside plants: bacterial endophytes. Curr. Opin. Plant Biol. 14, 435–443. 10.1016/j.pbi.2011.04.004 21536480

[B49] Richter-Heitmann T. Eickhorst T. Knauth S. Friedrich M. W. Schmidt H. (2016). Evaluation of strategies to separate root-associated microbial communities: a crucial choice in rhizobiome research. Front. Microbiol. 7, 773. 10.3389/fmicb.2016.00773 27252690 PMC4877504

[B50] Riva V. Mapelli F. Bagnasco A. Mengoni A. Borin S. (2022). A meta-analysis approach to defining the culturable core of plant endophytic bacterial communities. Appl. Environ. Microbiol. 88, e02537–e02521. 10.1128/aem.02537-21 35138928 PMC8939329

[B51] Rodriguez P. A. Rothballer M. Chowdhury S. P. Nussbaumer T. Gutjahr C. Falter-Braun P. (2019). Systems biology of plant microbiome interactions. Mol. Plant 12, 804–821. 10.1016/j.molp.2019.05.006 31128275

[B52] Rosenblueth M. Martínez-Romero E. (2006). Bacterial endophytes and their interactions with hosts. Mol. Plant-Microbe Int. 19, 827–837. 10.1094/MPMI-19-0827 16903349

[B53] Ryan R. P. Germaine K. Franks A. Ryan D. J. Dowling D. N. (2008). Bacterial endophytes: recent developments and applications. FEMS Microbiol. Lett. 278, 1–9. 10.1111/j.1574-6968.2007.00918.x 18034833

[B54] Sánchez-López A. S. Thijs S. Beckers B. González-Chávez M. Weyens N. Carrillo-González R.. (2018). Community structure and diversity of endophytic bacteria in seeds of three consecutive generations of *Crotalaria pumila* growing on metal mine residues. Plant Soil 422, 51–66. 10.1007/s11104-017-3176-2

[B55] Sarhan M. S. Hamza M. A. Youssef H. H. Patz S. Becker M. Elsawey H.. (2019). Culturomics of the plant prokaryotic microbiome and the dawn of plant-based culture media—A review. J. Adv. Res. 19, 15–27. 10.1016/j.jare.2019.04.002 31341666 PMC6630032

[B56] Shakya M. Gottel N. Castro H. Yang Z. K. Gunter L. Labbé J.. (2013). A multifactor analysis of fungal and bacterial community structure in the root microbiome of mature *Populus deltoides* trees. PLoS ONE 8, e76382. 10.1371/journal.pone.0076382 24146861 PMC3797799

[B57] Shehata H. R. Ragupathy S. Henry T. A. Newmaster S. G. (2021). Niche specificity and functional diversity of the bacterial communities associated with Ginkgo biloba and Panax quinquefolius. Sci. Rep. 11, 10803. 10.1038/s41598-021-90309-0 34031502 PMC8144622

[B58] Singh R. Pandey K. D. Singh M. Singh S. K. Hashem A. Al-Arjani A.-B. F.. (2022). Isolation and characterization of endophytes bacterial strains of *Momordica charantia* L. and their possible approach in stress management. Microorganisms 10, 290. 10.3390/microorganisms10020290 35208743 PMC8877101

[B59] Stewart Eric J. (2012). Growing unculturable bacteria. J. Bacteriol. 194, 4151–4160. 10.1128/JB.00345-12 22661685 PMC3416243

[B60] Toghueo R. M. K. (2020). Endophytes from *Gingko biloba*: the current status. Phytochem. Rev. 19, 743–759. 10.1007/s11101-020-09679-4

[B61] Utturkar S. M. Cude W. N. Robeson Jr M. S. Yang Z. K. Klingeman D. M. Land M. L.. (2016). Enrichment of root endophytic bacteria from *Populus deltoides* and single-cell-genomics analysis. Appl. Environ. Microbiol. 82, 5698–5708. 10.1128/AEM.01285-16 27422831 PMC5007785

[B62] Wang Y. Liu Y. Wu Q. Yao X. Cheng Z. (2017). Rapid and sensitive determination of major active ingredients and toxic components in *Ginkgo biloba* leaves extract (EGb 761) by a validated UPLC–MS-MS method. J. Chromatogr. Sci. 55, 459–464. 10.1093/chromsci/bmw206 28069691

[B63] Wemheuer F. Taylor J. A. Daniel R. Johnston E. Meinicke P. Thomas T.. (2020). Tax4Fun2: prediction of habitat-specific functional profiles and functional redundancy based on 16S rRNA gene sequences. Environ. Microbiome 15, 1–12. 10.1186/s40793-020-00358-7 33902725 PMC8067651

[B64] Yuan Z. Tian Y. He F. Zhou H. (2019). Endophytes from *Ginkgo biloba* and their secondary metabolites. Chin. Med-UK 14, 1–40. 10.1186/s13020-019-0271-8 31728156 PMC6842171

[B65] Zhang J. Liu Y.-X. Zhang N. Hu B. Jin T. Xu H.. (2019). *NRT1.1B* is associated with root microbiota composition and nitrogen use in field-grown rice. Nat. Biotechnol. 37, 676–684. 10.1038/s41587-019-0104-4 31036930

[B66] Zhang J. Zhang N. Liu Y.-X. Zhang X. Hu B. Qin Y.. (2018). Root microbiota shift in rice correlates with resident time in the field and developmental stage. Sci. China Life Sci. 61, 613–621. 10.1007/s11427-018-9284-4 29582350

[B67] Zhang Y. Zhang T. (2022). Culturing the uncultured microbial majority in activated sludge: a critical review. Crit. Rev. Environ. Sci. Technol. 6, 1–24. 10.1080/10643389.2022.2077063

[B68] Zhao Y.-P. Fan G. Yin P.-P. Sun S. Li N. Hong X.. (2019). Resequencing 545 *ginkgo* genomes across the world reveals the evolutionary history of the living fossil. Nat. Commun. 10, 4201. 10.1038/s41467-019-12133-5 31519986 PMC6744486

[B69] Zou K. Liu X. Hu Q. Zhang D. Fu S. Zhang S.. (2021). Root endophytes and *Ginkgo biloba* are likely to share and compensate secondary metabolic processes, and potentially exchange genetic information by LTR-RTs. Front. Plant Sci. 12, 704985. 10.3389/fpls.2021.704985 34305992 PMC8301071

