# Supplementary material for: Enriching the endophytic bacterial microbiota of Ginkgo roots
Source: Front Microbiol. 2023 Apr 17;14:1163488. doi: 10.3389/fmicb.2023.1163488 (PMC10150934; doi:10.3389/fmicb.2023.1163488)
Supplement: Supplementary file 1 [file Data_Sheet_1.docx]

Supplementary Material

Enriching the endophytic bacterial microbiota of Ginkgo roots

Shuangfei Zhang, Chongran Sun, Xueduan Liu, Yili Liang*

*** Correspondence:** Corresponding Author: liangyili6@csu.edu.cn

# Supplementary Figures


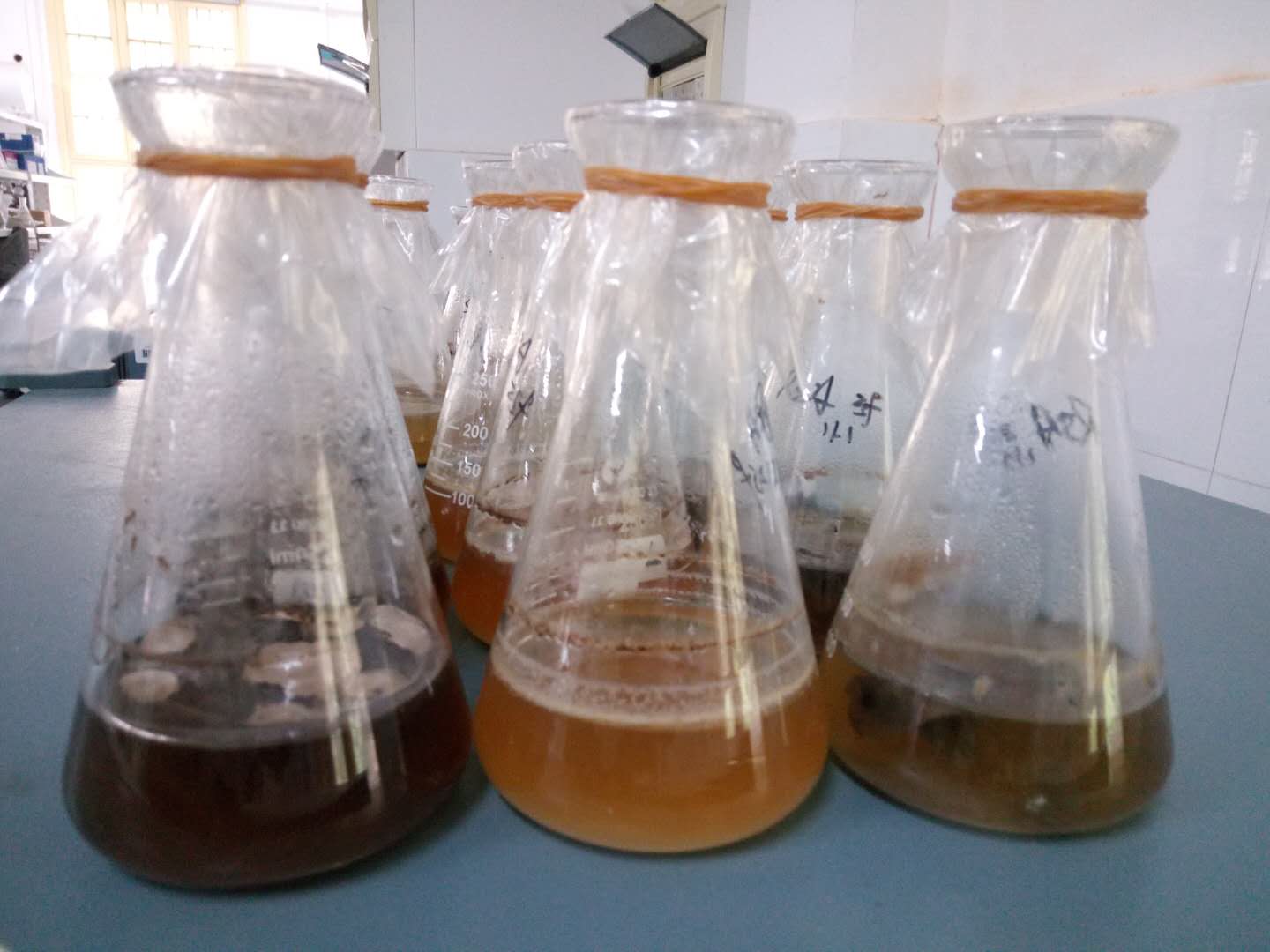


**Supplementary Figure 1.** The upper plot showed the mixed fermentative broth after the 8-days enrichment. Left flasks represent the fermentative broth using MM; middle flasks represent the fermentative broth using GM; right flasks represent the fermentative broth using MSM. Table 1 presents a list of the abbreviations of the groups.


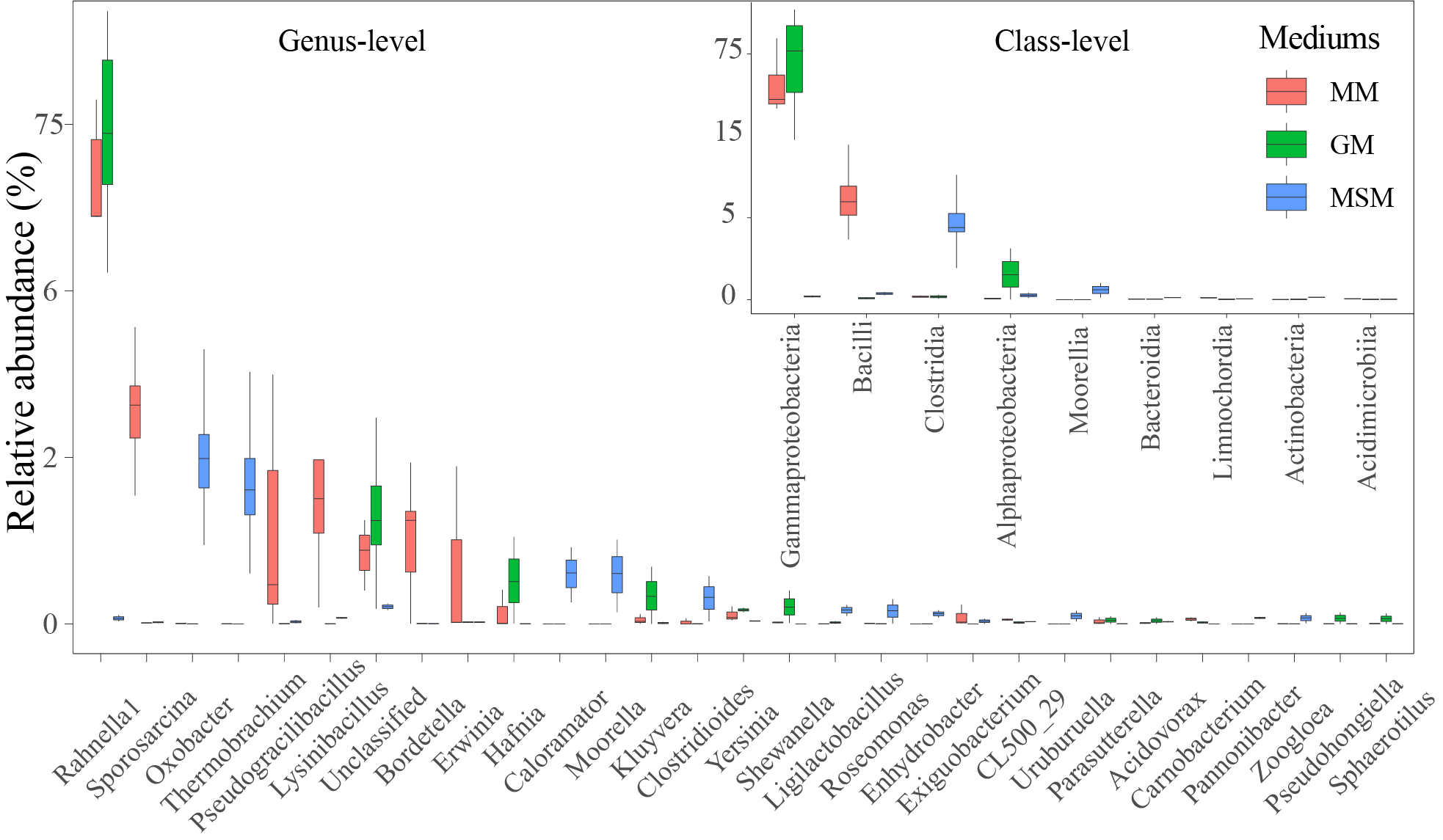


**Supplementary Figure 2.** Composition of specific bacterial taxa (913 OTUs) in the enrichment collections. The plots show the top 9 most abundant class and the top 29 most abundant genera of bacterial communities, respectively. Table 1 presents a list of the abbreviations of the groups.


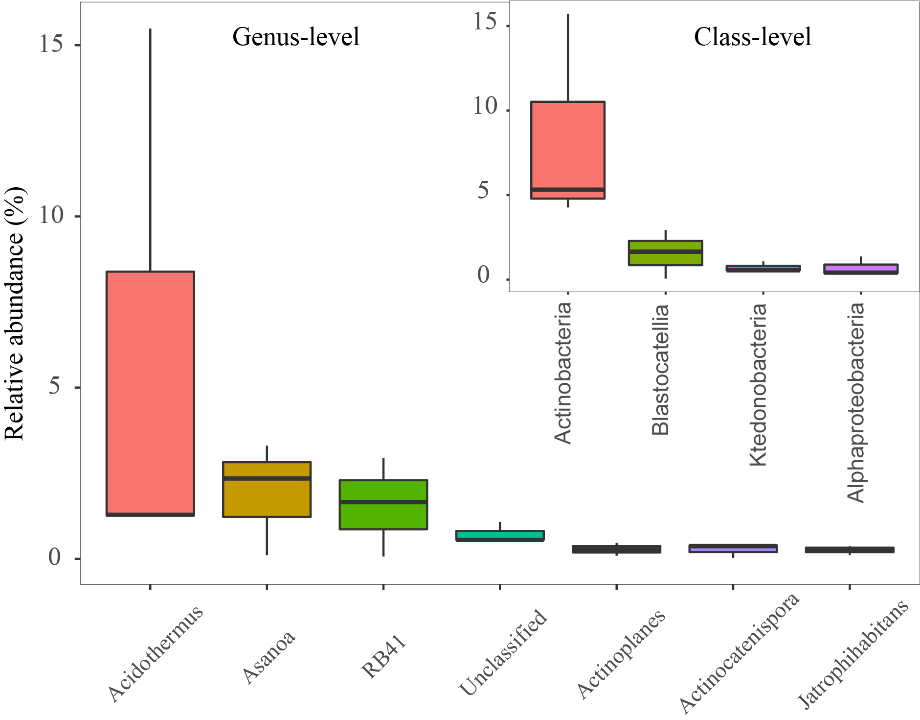


**Supplementary Figure 3.** Composition of specific bacterial taxa (245 OTUs) in the in-situ root samples. The plots show the top 4 most abundant class and the top 7 most abundant genera of bacterial communities, respectively. Table 1 presents a list of the abbreviations of the groups.


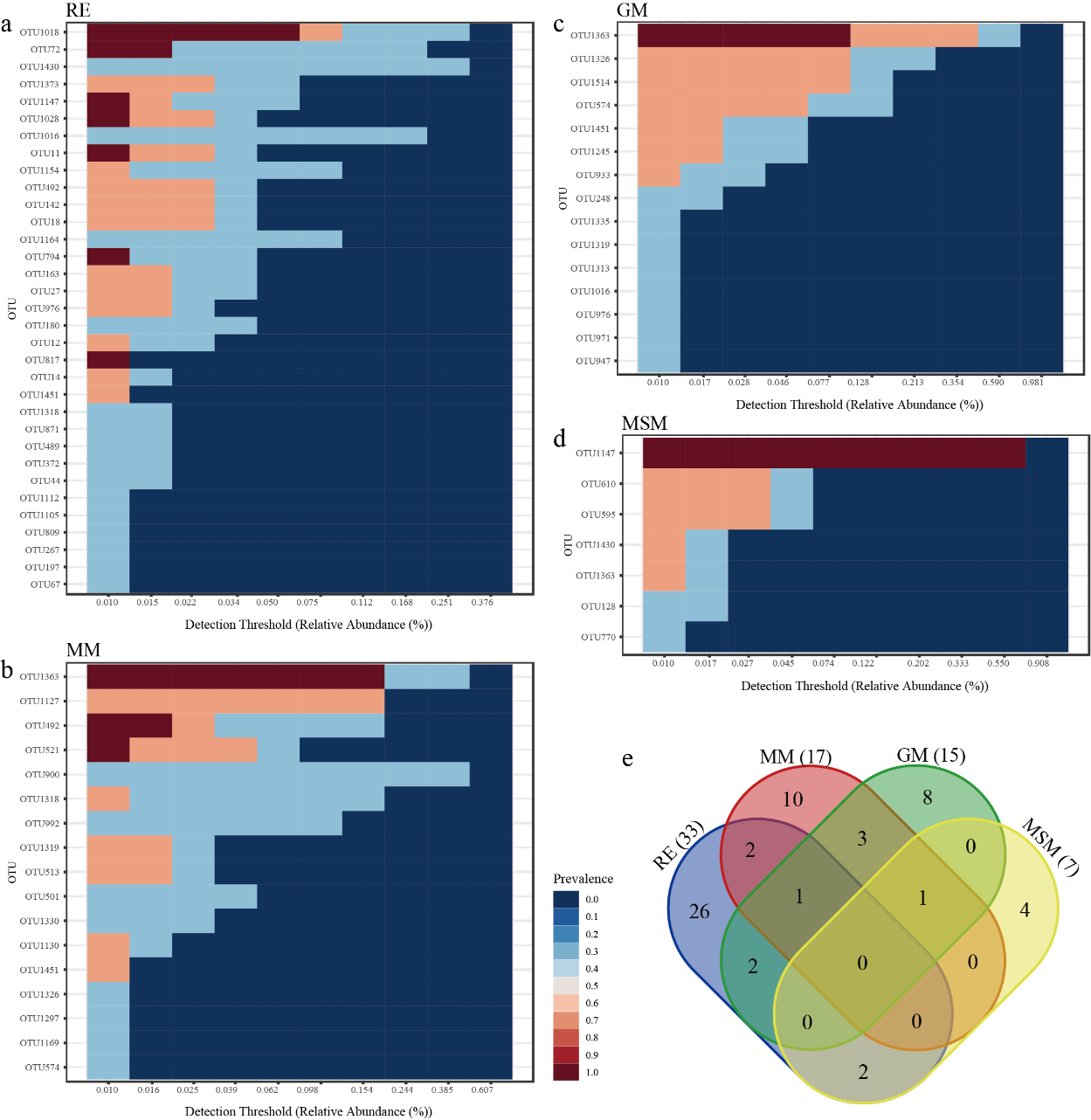


**Supplementary Figure 4.** Bacterial core microbes at OTU level. Table 1 presents a list of the abbreviations of the groups.


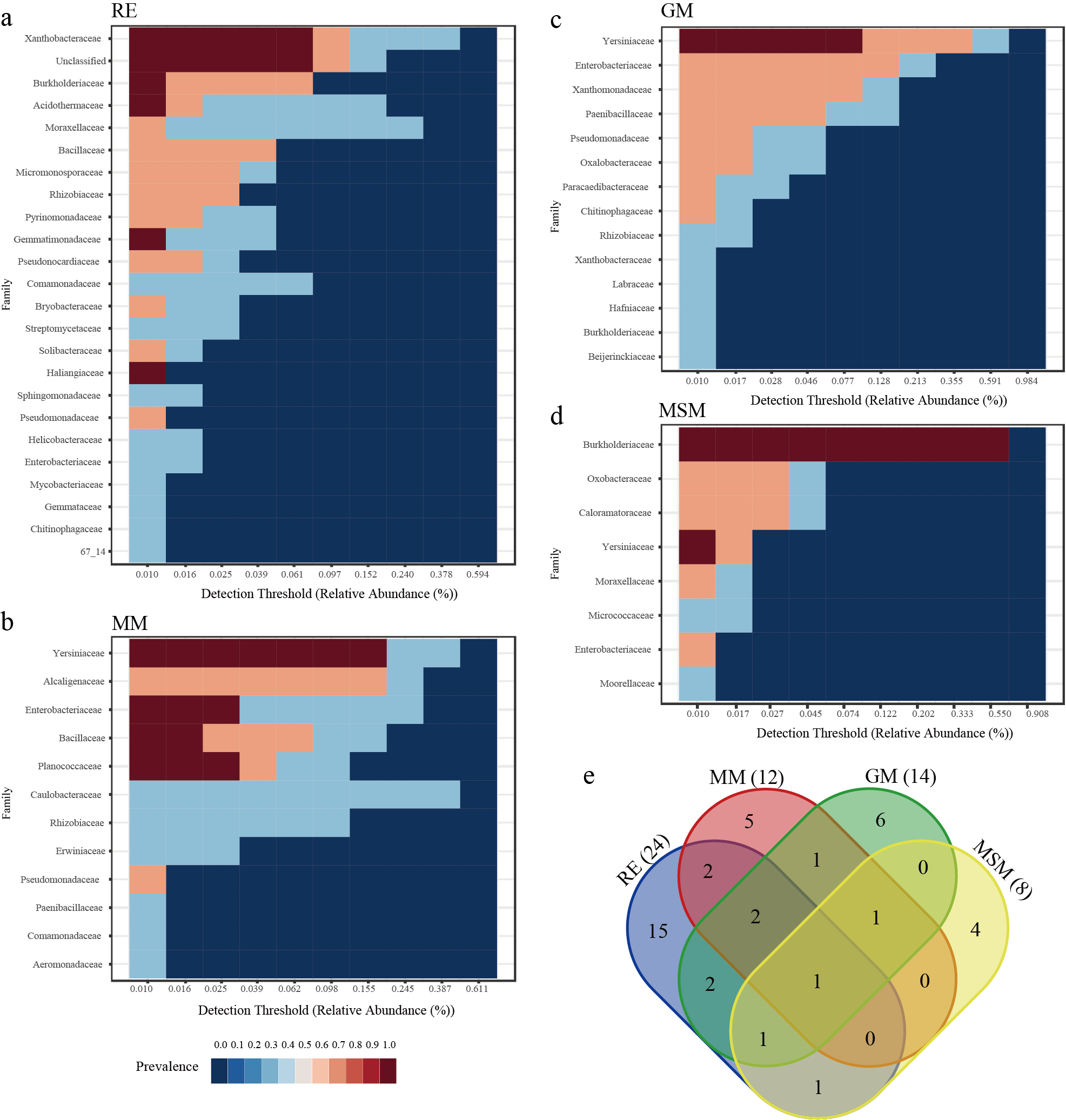


**Supplementary Figure 5.** Bacterial core microbes at family level. Table 1 presents a list of the abbreviations of the groups.


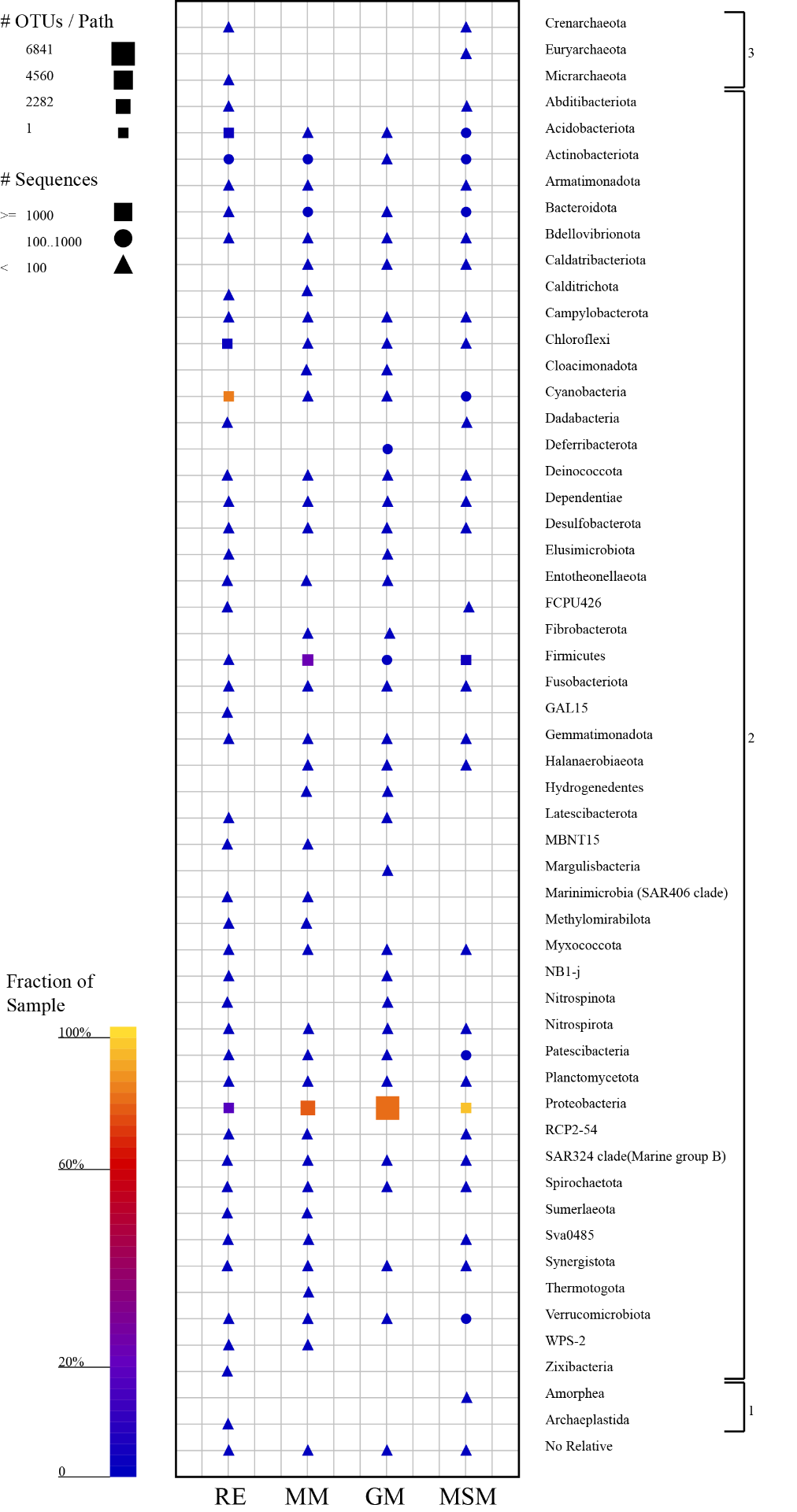


**Supplementary Figure 6.** Bacterial taxonomic fingerprint at phylum level. Table 1 presents a list of the abbreviations of the groups.


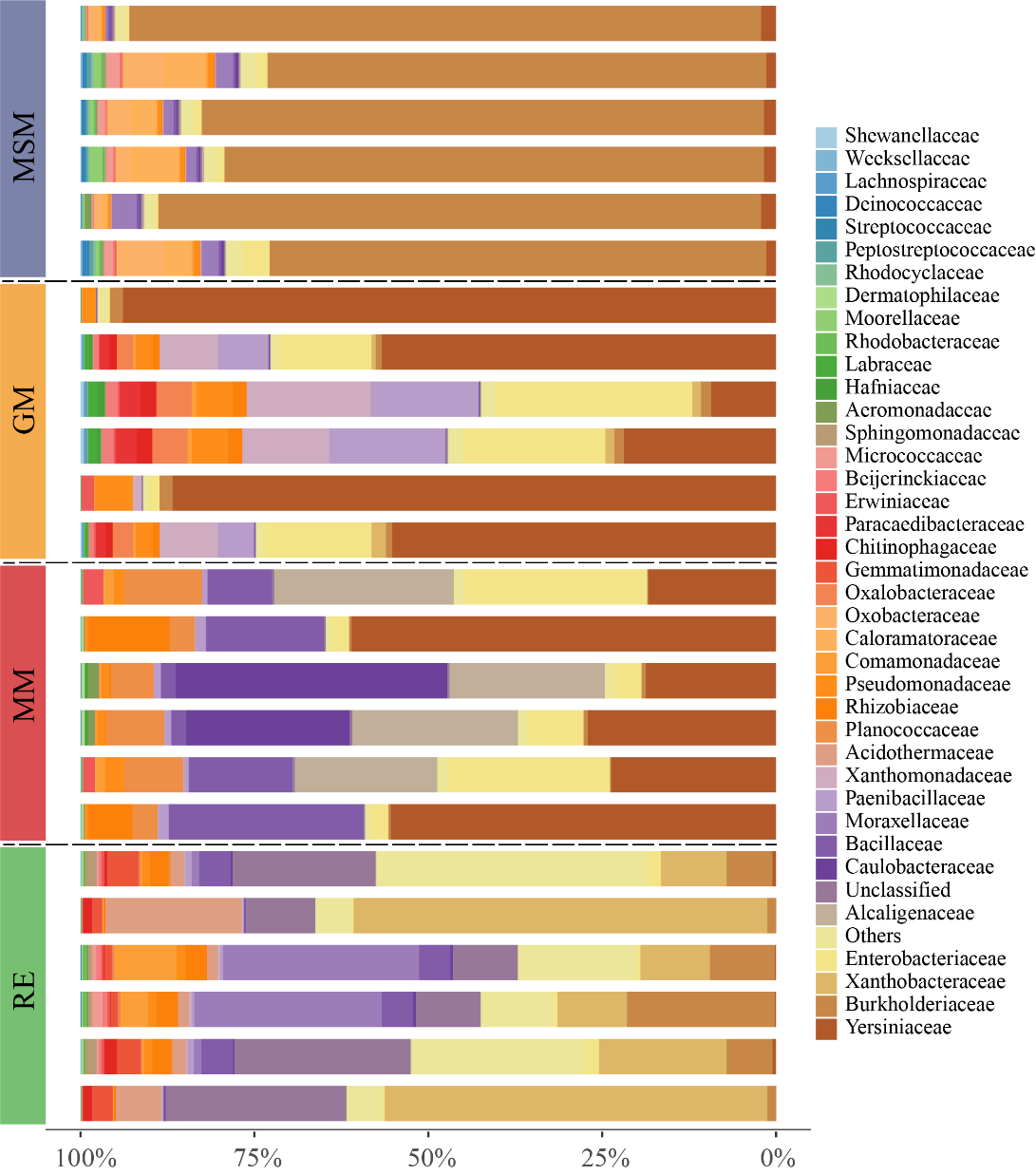


**Supplementary Figure 7.** Relative abundances of sequences at the family level. Groups with <500 reads were summarized as “Other.” Table 1 presents a list of the abbreviations of the groups.


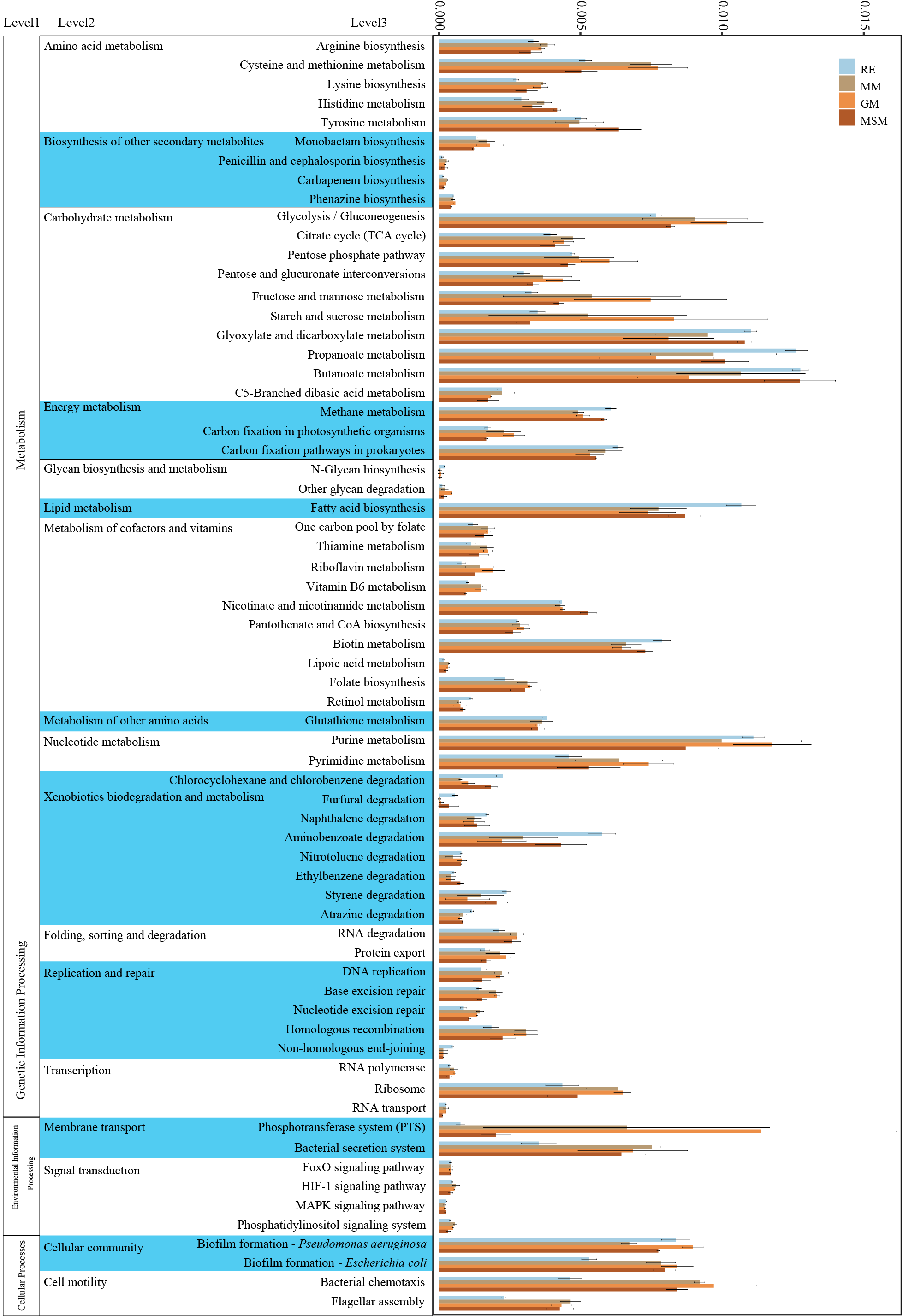


**Supplementary Figure 8.** Functional prediction of different bacterial communities based on the software Tax4Fun2. Table 1 presents a list of the abbreviations of the groups.


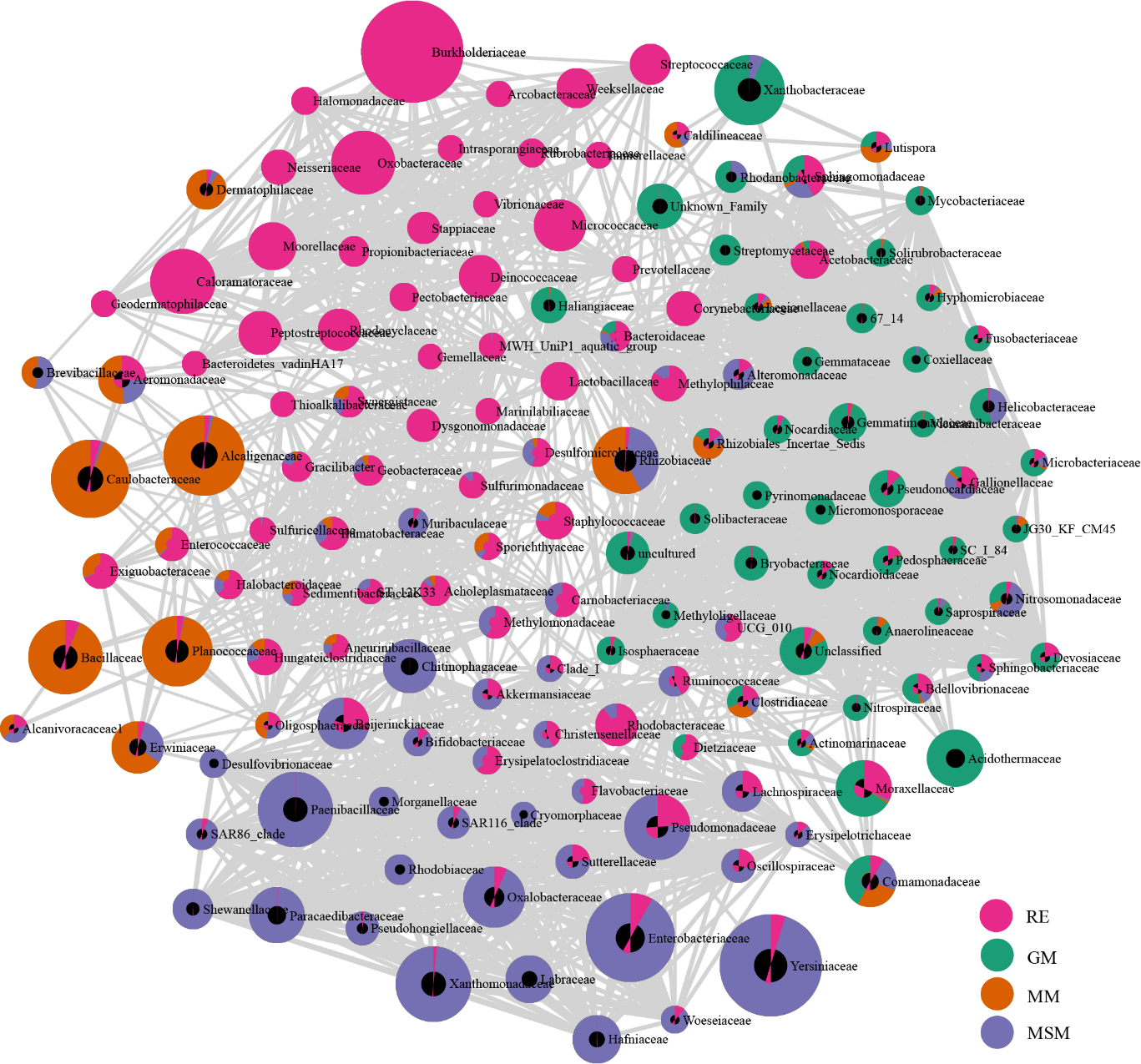


**Supplementary Figure 9.** Bacterial interactive network at family level. Table 1 presents a list of the abbreviations of the groups.
